# Ancient DNA suggests modern wolves trace their origin to a Late Pleistocene expansion from Beringia

**DOI:** 10.1111/mec.15329

**Published:** 2020-01-02

**Authors:** Liisa Loog, Olaf Thalmann, Mikkel‐Holger S. Sinding, Verena J. Schuenemann, Angela Perri, Mietje Germonpré, Herve Bocherens, Kelsey E. Witt, Jose A. Samaniego Castruita, Marcela S. Velasco, Inge K. C. Lundstrøm, Nathan Wales, Gontran Sonet, Laurent Frantz, Hannes Schroeder, Jane Budd, Elodie‐Laure Jimenez, Sergey Fedorov, Boris Gasparyan, Andrew W. Kandel, Martina Lázničková‐Galetová, Hannes Napierala, Hans‐Peter Uerpmann, Pavel A. Nikolskiy, Elena Y. Pavlova, Vladimir V. Pitulko, Karl‐Heinz Herzig, Ripan S. Malhi, Eske Willerslev, Anders J. Hansen, Keith Dobney, M. Thomas P. Gilbert, Johannes Krause, Greger Larson, Anders Eriksson, Andrea Manica

**Affiliations:** ^1^ Research Laboratory for Archaeology and History of Art University of Oxford Oxford UK; ^2^ Department of Zoology University of Cambridge Cambridge UK; ^3^ Manchester Institute of Biotechnology School of Earth and Environmental Sciences University of Manchester Manchester UK; ^4^ Department of Genetics University of Cambridge Cambridge UK; ^5^ Department of Pediatric Gastroenterology and Metabolic Diseases Poznan University of Medical Sciences Poznan Poland; ^6^ EvoGenomics GLOBE Institute University of Copenhagen Copenhagen Denmark; ^7^ Natural History Museum University of Oslo Oslo Norway; ^8^ The Qimmeq Project University of Greenland Nuussuaq Greenland; ^9^ Institute for Archaeological Sciences University of Tübingen Tübingen Germany; ^10^ Senckenberg Centre for Human Evolution and Palaeoenvironment University of Tübingen Tübingen Germany; ^11^ Institute of Evolutionary Medicine University of Zurich Zurich Switzerland; ^12^ Department of Human Evolution Max Planck Institute for Evolutionary Anthropology Leipzig Germany; ^13^ OD Earth and History of Life Royal Belgian Institute of Natural Sciences Brussels Belgium; ^14^ Department of Geosciences, Palaeobiology University of Tübingen Tübingen Germany; ^15^ School of Integrative Biology University of Illinois at Urbana‐Champaign Urbana IL USA; ^16^ BioArch, Department of Archaeology University of York York UK USA; ^17^ OD Taxonomy and Phylogeny Royal Belgian Institute of Natural Sciences Brussels Belgium; ^18^ Breeding Centre for Endangered Arabian Wildlife Sharjah United Arab Emirates; ^19^ Mammoth Museum Institute of Applied Ecology of the North of the North‐Eastern Federal University Yakutsk Russia; ^20^ Institute of Archaeology and Ethnography National Academy of Sciences of the Republic of Armenia Yerevan Republic of Armenia; ^21^ Heidelberg Academy of Sciences and Humanities: The Role of Culture in Early Expansions of Humans Tübingen Germany; ^22^ Department of Anthropology University of West Bohemia Pilzen Czech Republic; ^23^ Moravian museum Brno Czech Republic; ^24^ Hrdlička Museum of Man Faculty of Science Charles University Praha Czech Republic; ^25^ Institute of Palaeoanatomy Domestication Research and History of Veterinary Medicine Ludwig‐Maximilians‐University Munich Munich Germany; ^26^ Geological Institute Russian Academy of Sciences Moscow Russia; ^27^ Institute for Material Culture History Russian Academy of Sciences St Petersburg Russia; ^28^ Arctic and Antarctic Research Institute St Petersburg Russia; ^29^ Institute of Biomedicine and Biocenter of Oulu Medical Research Center and University Hospital University of Oulu Oulu Finland; ^30^ Carl R. Woese Institute for Genomic Biology University of Illinois at Urbana‐Champaign Urbana IL USA; ^31^ Centre for GeoGenetics Globe Institute University of Copenhagen Copenhagen Denmark; ^32^ Wellcome Trust Sanger Institute Cambridge UK; ^33^ Department of Archaeology, Classics and Egyptology University of Liverpool Liverpool UK; ^34^ Department of Archaeology University of Aberdeen Aberdeen UK; ^35^ Department of Archaeology Simon Fraser University Burnaby BC Canada; ^36^ Norwegian University of Science and Technology University Museum Trondheim Norway; ^37^ Max Planck Institute for the Science of Human History Jena Germany; ^38^ Department of Medical & Molecular Genetics King's College London Guys Hospital London UK; ^39^ cGEM, Institute of Genomics, University of Tartu Tartu Estonia

**Keywords:** Approximate Bayesian Computation, ancient DNA, coalescent modelling, megafauna, Pleistocene, population structure, population turnover, wolves

## Abstract

Grey wolves (*Canis lupus*) are one of the few large terrestrial carnivores that have maintained a wide geographical distribution across the Northern Hemisphere throughout the Pleistocene and Holocene. Recent genetic studies have suggested that, despite this continuous presence, major demographic changes occurred in wolf populations between the Late Pleistocene and early Holocene, and that extant wolves trace their ancestry to a single Late Pleistocene population. Both the geographical origin of this ancestral population and how it became widespread remain unknown. Here, we used a spatially and temporally explicit modelling framework to analyse a data set of 90 modern and 45 ancient mitochondrial wolf genomes from across the Northern Hemisphere, spanning the last 50,000 years. Our results suggest that contemporary wolf populations trace their ancestry to an expansion from Beringia at the end of the Last Glacial Maximum, and that this process was most likely driven by Late Pleistocene ecological fluctuations that occurred across the Northern Hemisphere. This study provides direct ancient genetic evidence that long‐range migration has played an important role in the population history of a large carnivore, and provides insight into how wolves survived the wave of megafaunal extinctions at the end of the last glaciation. Moreover, because Late Pleistocene grey wolves were the likely source from which all modern dogs trace their origins, the demographic history described in this study has fundamental implications for understanding the geographical origin of the dog.

## Abstract 2

see also the Perspective by Rena M. Schweizer and Robert K. Wayne.

## 1 INTRODUCTION

The Pleistocene epoch harboured a large diversity of top predators, although most became extinct during or soon after the Last Glacial Maximum (LGM), ~21,000 years ago (Barnosky, Koch, Feranec, Wing, & Shabel, 2004; Clark et al., 2012). The grey wolf (*Canis lupus*) was one of the few large carnivores that survived and maintained a wide geographical range throughout the period (Puzachenko & Markova, 2016), and both the palaeontological and archaeological records attest to the continuous presence of grey wolves across the Northern Hemisphere for at least the last 300,000 years (Sotnikova & Rook, 2010) (reviewed in Appendix S1). This geographical and temporal continuity across the Northern Hemisphere contrasts with analyses of complete modern genomes, which have suggested that all contemporary wolves and dogs descend from a common ancestral population that existed as recently as 20,000 years ago (Fan et al., 2016; Freedman et al., 2014; Skoglund, Ersmark, Palkopoulou, & Dalén, 2015).

These analyses point to a bottleneck followed by a rapid radiation from an ancestral population around or just after the LGM. The geographical origin and dynamics of this radiation remain unknown. Resolving these demographic changes is necessary for understanding the ecological circumstances that allowed wolves to survive the Late Pleistocene megafaunal extinctions. Furthermore, because dogs were domesticated from Late Pleistocene grey wolves (Larson et al., 2012), a detailed insight into wolf demography during this time period would provide an essential context for reconstructing the history of dog domestication.

Reconstructing past demographic events solely from modern genomes is challenging because multiple demographic histories can lead to similar genetic patterns in present‐day samples (Groucutt et al., 2015). Analyses that incorporate ancient DNA sequences can eliminate some of these alternative histories by quantifying changes in population genetic differences through time. While nuclear markers provide greater power relative to mitochondrial DNA (mtDNA), the latter is more easily retrievable and better preserved in ancient samples due to its higher copy number compared to nuclear DNA, thus allowing for the generation of data sets with greater geographical and temporal coverage. In particular, analysing samples dated to before, during and after the demographic events of interest greatly increases the power to infer past demographic histories. Furthermore, the nuclear mutation rate in canids is poorly understood, leading to wide date ranges for past demographic events reconstructed from panels of modern whole genomes (e.g., Fan et al., 2016; Freedman et al., 2014). Having directly dated samples from a broad time period allows us to estimate mutation rates with higher accuracy and precision compared to alternative methods (Drummond, Nicholls, Rodrigo, & Solomon, 2002; Rambaut, 2000; Rieux et al., 2014).

Demographic processes, such as range expansions and contractions, that involved space as well as time are particularly challenging to reconstruct as they often lead to patterns that are difficult to interpret intuitively (Groucutt et al., 2015). Hypotheses involving spatial processes can be formally tested using population genetic models that explicitly represent the various demographic processes and their effect on genetic variation through time and across space (Eriksson et al., 2012; Eriksson & Manica, 2012; Posth et al., 2016; Raghavan et al., 2015; Warmuth et al., 2012). The formal integration of time and space into population genetics frameworks allows for the analysis of sparse data sets, a common challenge when dealing with ancient DNA (Loog et al., 2017).

**Figure 1:**
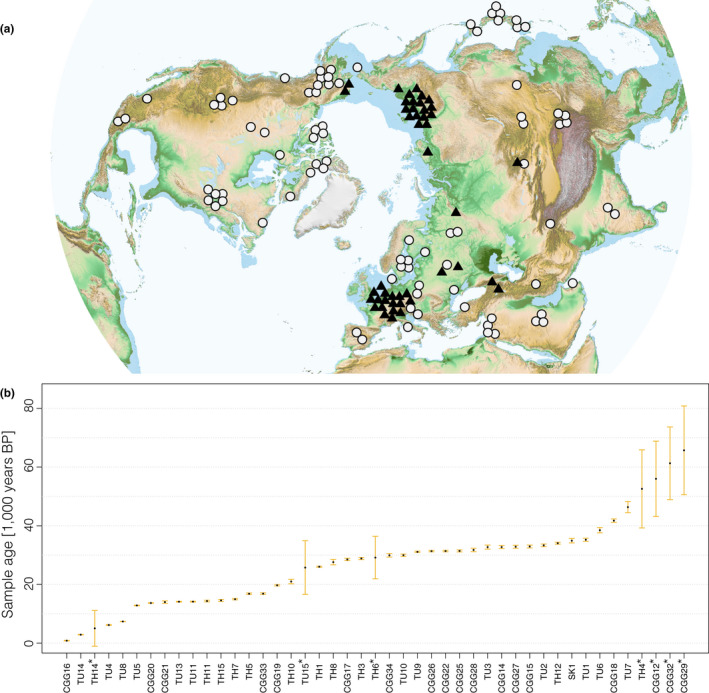
Geographical distribution of modern (<500 years old, circles) and ancient (>500 years old, triangles) samples (a) and temporal distribution of ancient samples (b) used in the analyses. The geographical locations of the samples have been slightly adjusted for clarity (see Table S1 for exact sample locations). *Samples dated by molecular dating [Colour figure can be viewed at wileyonlinelibrary.com]

Here, we use a spatially explicit population genetic framework to model a range of different demographic histories of wolves across the Northern Hemisphere that involve combinations of population bottlenecks, turnover and long‐range migrations as well as local gene flow. To estimate model parameter and formally test hypotheses of the origin and population dynamics of the expansion of grey wolves during the LGM, we assembled a substantial data set (Figure 1; Table S1), spanning the last 50,000 years and the geographical breadth of the Northern Hemisphere. This data set consists of 90 modern and 45 ancient wolf whole mitochondrial genomes (38 of which are newly sequenced). In the following, we first present a phylogenetic analysis of our sequences and a calibration of the wolf mitochondrial mutation rates. We then perform formal hypothesis testing using Approximate Bayesian Computation (ABC) with our spatiotemporally explicit models. We conclude with a discussion of how our findings relate to earlier studies and implications for future research.

## 2 MATERIALS AND METHODS

### 2.1 Data preparation

We sequenced whole mitochondrial genomes of 40 ancient wolf samples. Sample information, including geographical locations, estimated ages and archaeological context information for the ancient samples, is provided in Table S1 and Appendix S1. Of the 40 ancient samples, 24 were directly radiocarbon dated for this study and calibrated using the IntCal13 calibration curve (see Table S1 for radiocarbon dates, calibrated age ranges and accelerator mass spectrometry [AMS] laboratory reference numbers). DNA extraction, sequencing and quality filtering, and mapping protocols used are described in Appendix S2.

We included 16 previously published ancient mitochondrial wolf genomes (Table S1 and Appendix S2). To achieve a uniform data set, we reprocessed the raw reads from previously published samples using the same bioinformatics pipeline as for the newly generated sequences.

We subjected the aligned ancient sequences to strict quality criteria in terms of damage patterns and missing data (Figures S3–S5). First, we excluded all whole mitochondrial sequences that had more than one‐third of the whole mitochondrial genome missing (excluding the mitochondrial control region—see below) at minimum three‐fold coverage. Second, we excluded all ancient whole mitochondrial sequences that contained more than 0.1% of singletons showing signs of deamination damage typical for ancient DNA (C to T or A to G singletons). After quality filtering, we were left with 32 newly sequenced and 13 published ancient whole mitochondrial sequences (Table S1).

We also excluded sequences from archaeological specimens that post‐date the end of the Pleistocene and that have been identified as dogs (Table S1), because any significant population structure resulting from a lack of gene flow between dogs and wolves could violate the assumption of a single, randomly mating canid population. Some of the Pleistocene specimens used in the demographic analyses (TH5, TH12, TH14) have been argued to show features commonly found in modern dogs and have therefore been suggested to represent Palaeolithic dogs (e.g., Druzhkova et al., 2013; Germonpré, Lázničková‐Galetová, Losey, Räikkönen, & Sablin, 2015; Germonpré, Lázničková‐Galetová, & Sablin, 2012; Germonpré et al., 2009; Sablin & Khlopachev, 2002). Here, we disregard such status calls because of the controversy that surrounds them (Crockford & Kuzmin, 2012; Drake, Coquerelle, & Colombeau, 2015; Morey, 2014; Perri, 2016), and because early dogs would have been genetically similar to the local wolf populations form which they derived. This reasoning is supported by the close proximity of these samples to other wolf specimens confidently described as wolves in the phylogenetic tree (see Figure S10).

Finally, we sequenced six samples from modern wolves and added 66 modern published wolf sequences from NCBI, two sequences from Freedman et al. (2014), 13 sequences from Sinding et al. (2018) and three sequences from Gopalakrishnan et al. (2018) (Table S1). Data from Sinding et al. (2018) and Gopalakrishnan et al. (2018) were newly assembled following the same bioinformatics protocols as were used for newly sequenced modern wolf samples (see Appendix S2). This resulted in a final data set of 135 complete wolf mitochondrial genome sequences, of which 45 were ancient and 90 were modern. We used the clustalw alignment tool (version 2.1) (Larkin et al., 2007) to generate a joint alignment of all genomes. To avoid the potentially confounding effect of recurrent mutations in the mitochondrial control region (Excoffier & Yang, 1999) in pairwise difference calculations, we removed this region from all subsequent analyses. This resulted in an alignment of sequences 15,466 bp in length, of which 1,301 sites (8.4%) were variable. The aligned data set is given in Appendix S1.

### 2.2 Phylogenetic analysis

We calculated the number of pairwise differences between all samples (Figure S6) and generated a neighbour‐joining tree based on pairwise differences (Figure S7). This tree shows a clade consisting of samples exclusively from the Tibetan region and the Indian subcontinent that are deeply diverged from all ancient and other modern wolf samples (see also Aggarwal, Kivisild, Ramadevi, & Singh, 2007; Sharma, Maldonado, Jhala, & Fleischer, 2004). A recent study of whole genome data showed a complex history of South Eurasian wolves (Fan et al., 2016) that is beyond the scope of our study. While their neighbour‐joining phylogeny grouped South Eurasian wolves with East and North East Asian wolves (Fan et al., 2016: Figure 3), they cluster outside of all other grey wolves in a principal component analysis (Fan et al., 2016: Figure 4), and also show a separate demographic history within a Pairwise Sequentially Markovian Coalescent analysis (PSMC) (Fan et al., 2016: Figure 5). Because our study did not possess sufficient samples from the Himalayas and the Indian subcontinent to unravel their complex demography, we excluded samples from these regions and focused on the history of North Eurasian and North American wolves, for which we have good coverage through time and space.

We used partitionfinder (Lanfear, Calcott, Ho, & Guindon, 2012) and beast (version 1.8.0) (Drummond, Suchard, Xie, & Rambaut, 2012) to build a tip calibrated wolf mitochondrial tree (with a strict global clock, see Appendix S1 for full details) from modern and directly dated ancient samples, and to estimate mutation rates for four different partitions of the wolf mitochondrial genome (see Tables S3 and S4 for results).

We used beast to molecularly date seven sequences from samples that were not directly radiocarbon dated (TH4, TH6, TH14, TU15) or that had been dated to a period beyond the limit of reliable radiocarbon dating (>48,000 years ago) (CGG12, CGG29, CGG32). We estimated the ages of the samples by performing a beast run where the mutation rate was fixed to the mean estimates from the previous beast analysis and all other parameter settings were set as described in Appendix S1. We cross‐validated this approach through a leave‐one‐out analysis where we sequentially removed a directly dated sample and estimated its date as described above. We find a close fit (*R* ^2^ = 0.86) between radiocarbon and molecular dates (Figure S9). We combined the seven undated samples with the 110 ancient and modern samples from the previous run and used a uniform prior ranging from 0 to 100,000 years to estimate the ages of the seven undated samples (see Table S5 for results).

Finally, in order to estimate the mitochondrial divergence time between the South Eurasian (Tibetan and Indian) and the rest of our wolf samples, we performed an additional beast run in which we included all modern and ancient grey wolves (*N* = 129) as well as five Tibetan and one Indian wolf, and used parameters identical to those described above. The age of the ancient samples was set as the mean of the calibrated radiocarbon date distribution (for radiocarbon‐dated samples) or as the mean of the age distribution from the beast analyses (for molecularly dated samples).

### 2.3 Isolation by distance analysis

We performed isolation by distance (IBD) analyses to see the extent to which wolf mitochondrial genetic variation shows population structure. To this end, we regressed the pairwise geographical distances between 84 modern wolf samples (Table S1) against their pairwise genetic (mitochondrial) distances. The geographical distance between all sample pairs was calculated in kilometres as the great circle distance from geographical coordinates, using the Haversine formula (Sinnott, 1984) to account for the curvature of the Earth as follows:

(1) $$G_{\mathrm{ij}}=2r\;\arcsin\left(\sqrt{\sin{\left(\left(\varphi_{j}-\varphi_{i}\right)/2\right)}^{2}+\cos\left(\varphi_{i}\right)\cos\left(\varphi_{j}\right)\sin{\left(\left(\lambda_{i}-\lambda_{j}\right)/2\right)}^{2}}\right)$$

where *G* is the distance in kilometres between individuals *i* and *j*; *φ_i_* and *φ_j_* are the latitude coordinates of individuals *i* and *j*, respectively; *λ_i_* and *λ_j_* are the longitude coordinates of individuals *i* and *j*, respectively; and *r* is the radius of the earth in kilometres. The pairwise genetic distances were calculated as the proportion of sites that differ between each pair of sequences (excluding the missing bases), using *dist.dna* function in the R package ape (Paradis, Claude, & Strimmer, 2004).

### 2.4 Geographical deme definitions

We represented the wolf geographical range as seven demes, defined by major geographical barriers through time.

1. The *European* deme is bordered by open water from the north and the west (the Arctic and the Atlantic oceans, respectively); the Ural Mountains from the east; and the Mediterranean, the Black and the Caspian Sea and the Caucasus mountains from the south.
2. The *Middle‐Eastern* deme consists of the Arabian Peninsula, Anatolia and Mesopotamia and is bordered by the Black Sea, the Caspian Sea and the Aral Sea in the north; the Indian Ocean in the south; the Tien Shen mountain range, the Tibetan Plateau and the Himalayas from the east; and the Mediterranean Sea in the west.
3. The *Central North Eurasian* deme consist of the Siberian Plateau and is bordered by the Arctic Ocean from the north; the Ural Mountains from the west; the Lena River and mountain ranges of northeastern Siberia (Chersky and Verkhoyansk ranges) from the east; and the Tien Shen mountain range, the Tibetan Plateau and the Gobi Desert from southeast.
4. The *East Eurasian *deme is bordered by the Tien Shen mountain range, the Tibetan Plateau and Gobi desert from the west; the Pacific Ocean from the east; and the Lena river and the mountain ranges of northeastern Siberia (Chersky and Verkhoyansk ranges) from the north.
5. The *Beringia* deme spans the Bering Strait, which was a land bridge during large parts of the Late Pleistocene and the Early Holocene. It is bordered to the west by the Lena River and mountain ranges of northeastern Siberia (Chersky and Verkhoyansk ranges), and to the south and east by the extent of the Cordillerian and Laurentide ice sheets during the LGM.
6. The *Arctic North America* deme consists of an area of the North American continent east of the Rocky Mountains and west of Greenland, that was covered by ice during the last glaciation and is at present known as the Canadian Arctic Archipelago.
7. The *North America* deme consists of an area in the Northern American subcontinent up to and including the area that was covered by the Cordillerian and Laurentide ice sheets during the last glaciation (Raghavan et al., 2015).

### 2.5 amovas

To quantify the extent that our geographical demes capture genetic variation in the data we performed analyses of molecular variance (amovas) (Excoffier, Smouse, & Quattro, 1992). We calculated the pairwise genetic distance between all modern wolf (*n* = 84, Table S1) sample pairs as described above (Isolation by distance analysis) and partitioned the samples, based on their geographical locations, into seven populations corresponding the geographical demes, as described in Section 2.4, Geographical deme definitions. We used these demes as the level of analyses and performed 1 million permutations using the *amova* function in the *R* package pegas (version 0.10). We found strong support for our geographical demes (*p* < 10^−6^) with 24.4% of the variance within the data set explained by the chosen demes.

### 2.6 Demographic scenarios

We tested a total of 16 demographic scenario combinations, from four different kinds of demographic scenarios (illustrated in Figure 4a):

1. Static model (the null hypothesis)—neighbouring demes exchange migrants, no demographic changes.
2. Bottleneck scenarios—demes exchange migrants as in the static model but populations have different size in different time periods. We consider three time periods: 0–15,000 years ago, 15,000–40,000 years ago, and >40,000 years ago.
3. Expansion scenarios—demes exchange migrants as in the static model but a single deme (which itself has a continuous population through time) experiences an expansion starting between 5,000 and 40,000 years ago (at a minimum rate of 1,000 years per deme, so the whole world could be colonized within 3,000 years or faster). The deme of origin has a continuous population through time while native populations in all other demes experience replacement—allowing us to formally test both the continuity and replacement hypotheses in each of the demes.
4. Combinations of scenarios 2 and 3.

### 2.7 Population genetic coalescent framework

We implemented coalescent population genetic models for the different demographic scenarios to sample gene genealogies.

In the static scenario, we simulated local coalescent processes (Kingman, 1982) within each deme (scaled to rate 1/*K* per pair of lineages, where *K* is the mean time to the most recent common ancestor (TMRCA) in a deme and is thus proportional to the effective population size). In addition, we moved lineages between demes according to a Poisson process with rate *m* per lineage. To match the geographical and temporal distribution of the data, we represented each sample with a lineage from the corresponding deme and date.

The bottleneck scenario was implemented as the static one but with piecewise constant values for *K* as a function of time. We considered three time periods, each with its own value of *K* (*K*_1_, *K*_2_ and *K*_3_), motivated by the archaeological and genetic evidence of wolf population changes described in the main text. The first time period was from the present to early Holocene, 0–15,000 years ago. The second time period extended from early Holocene to te Pleistocene and covered the LGM, 15,000–40,000 years ago. Finally, the third time period covered the Late Pleistocene and beyond, that is 40,000 years ago and older.

The population expansion scenarios were based on the static model but with an added population expansion model with founder effects and replacement of local populations (we refer to populations not yet replaced by the expansion as “indigenous”). Starting at time *T*, the population expanded from the initial deme and replaced its neighbouring populations. The population at the deme of origin was represented as a continuous population through time. After the start of the expansion, the expansion proceeded in fixed steps of Δ*T* (in time). At each step, colonized populations replaced neighbouring indigenous populations (if an indigenous deme bordered more than one colonized deme, these demes contributed equally to the colonization of the indigenous deme). In the coalescent framework (which simulates gene genealogies backwards in time) the colonization events corresponds to forced migrations from the indigenous deme to the source deme. If there were more than one source deme, the source of each lineage was chosen randomly with equal probability. Finally, founder effects during the colonization of an indigenous deme were implemented as a local, instantaneous population bottleneck in the deme (after the expansion), with a severity scaled to give a fixed probability *x* of a coalescent event for each pair of lineages in the deme during the bottleneck (Eriksson & Mehlig, 2004) (*x* = 1 corresponds to a complete loss of genetic diversity in the bottleneck, and *x* = 0 corresponds to no reduction in genetic diversity).

Finally, the combined scenario of population expansion and bottlenecks was implemented by making the population size parameter *K* in the population expansion model time‐dependent as in the population bottleneck model.

### 2.8 Approximate Bayesian Computation analysis

We used ABC analysis (Beaumont, Zhang, & Balding, 2002) with abctoolbox (Wegmann, Leuenberger, Neuenschwander, & Excoffier, 2010) to formally test the fit of our different demographic models. This approach allows formal hypothesis testing using likelihood ratios in the cases where the demographic scenarios are too complex for a direct calculation of the likelihoods given the models. We used the most likely tree from beast (see Appendix S1 for details) as data, and simulated trees using the coalescent simulations described above.

To match the assumption of random mixing within each deme in the population genetic model, we removed closely related sequences if they came from the same geographical location and time period, by randomly retaining one of the closely related sequences to be included in the analysis (Table S1, column “Samples_used_in_Simulation_Analysis”).

To robustly measure differences between simulated and observed trees we use the matrix of the TMRCA for all pairs of samples. This matrix also captures other allele frequency‐based quantities frequently used as summary statistics with ABC, such as *F*_ST_, as they can be calculated from the components of this matrix.

In principle the full matrix could be used, but in practice it is necessary to use a small number of summary statistics for ABC to work properly (Wegmann et al., 2010). To this end, we computed the mean TMRCA between pairs of sequences either within or between (a) Europe, (b) Middle East, (c) North East Eurasia, Beringia and East Eurasia combined; and (d) Artic and Continental North America combined. This strategy is based on geographical proximity and genetic similarity in the data set. We note that this is not the same as modelling the combined demes as a single panmictic deme; structure between the demes is still modelled explicitly, but the summary statistics are averaged over multiple demes.

An initial round of fitting the model showed that all scenarios underestimate the deme TMRCA for the Middle East, while the rest of the summary statistics were well captured by the best‐fitting demographic scenarios. This could be explained by a scenario where the Middle East was less affected by the reduction in population size during the LGM. However, we currently lack a sufficient number of samples from this area to explicitly test a more complex scenario such as this hypothesis. To avoid outliers biasing the likelihood calculations in ABC (Wegmann et al., 2010) we removed this summary statistic, resulting in nine summary statistics in total.

For each of the 16 scenarios we performed 1 billion simulations with randomly chosen parameter combinations, chosen from the following parameter intervals for the different scenarios:

- The static scenario: *m* in [0.001,20] and *K* in [0.01,100].
- The bottleneck scenarios: *m* in [0.001,20] and *K*_1_, *K*_2_, *K*_3_ in [0.01,100].
- The expansion scenarios: *m* in [0.001,20], *K* in [0.01,100], *x* in [0,1], *T* in [5,40] and Δ*T* in [0.001,1]. For expansion out of the North American scenario and expansion out of the Arctic North American scenario, glaciation during the LGM in North American and sea‐level rise during the deglaciation mean that *T* must be in the range [9,16]
- The combined bottleneck and expansion scenarios: *m* in [0.001,20], *K*_1_, *K*_2_, *K*_3_ in [0.01,100], *x* in [0,1], *T* in [5,40] and Δ*T* in [0.001,1].

The parameter *m* is measured in units of 1/1,000 years, and *T*, Δ*T*, *K*, *K*_1_, *K*_2_ and *K*_3_ are measured in units of 1,000 years. The parameters *x*, *T* and Δ*T* were sampled according to a uniform distribution over the interval, while all other parameters were sampled from a uniform distribution of their log‐transformed values. To identify good parameter combinations for ABC, we first calculated the Euclidian square distances between predicted and observed statistics and restricted analysis to parameter combinations within the lowest tenth distance percentile. We then ran the abctoolbox (Wegmann et al., 2010) on the accepted parameter combinations to estimate posterior distributions of the model parameters, and to calculate the likelihood of each scenario as described in the abctoolbox manual.

Table S6 provides ABC likelihoods and Bayes factors (BFs) for all demographic scenarios tested. Tables S7 and S8 give posterior probability estimates and Figures S13 and S14 give posterior density distributions for estimated parameters (Δ*T*, *T*, log_10_ *K*_1_, log_10_ *K*_2_, log_10_ *K*_3_, log_10_ *m*, *x*) in the two most likely models (an expansion out of Beringia with a population size change and an expansion out of East Eurasia with a population size change).

### 2.9 Map plots

The background map used in Figures 1(a) and 3(a), showing climatic regions on land masses, was generated by downloading the file color_etopo1_ice_low.jpg from ETOPO1 (Amante & Eakins, 2016), a one arc‐minute global relief model of the Earth's surface that integrates land topography and ocean bathymetry, and masking out regions where sea depths are greater than 120 m.

## 3 RESULTS

### 3.1 Population structure of grey wolf across the Northern Hemisphere

Motivated by the population structure observed in whole genome studies of modern wolves (Fan et al., 2016), we tested the degree of spatial genetic structure among the modern wolf samples in our data set, and found a strong pattern of genetic IBD across Eurasia (*ρ* = 0.3, *p *< .0001; see Figure S8). Ignoring this population structure (i.e., modelling wolves as a single panmictic population) can lead to artefactual results (Mazet, Rodríguez, & Chikhi, 2015; Mazet, Rodríguez, Grusea, Boitard, & Chikhi, 2016). The use of spatially structured models, in which migration is restricted to adjacent populations, is a common approach for dealing with such situations (Eriksson et al., 2012; Eriksson & Manica, 2012; Kimura & Weiss, 1964; Wegmann et al., 2010).

To capture the observed geographical structure in our data set, we split the Northern Hemisphere into seven regions, roughly similar in area (Figure 3a). The boundaries of these regions are defined by geographical features, including mountain ranges, seas, and deserts (see Materials and Methods), which are likely to reduce gene flow (Geffen, Anderson, & Wayne, 2004; Lucchini, Galov, & Randi, 2004) and provide an optimal balance between resolution and power given the distribution of samples available for analyses. To quantify how well this scheme represents population structure in modern wolves, we used an amova to separate genetic variance within and between regions. Our regions capture 24.4% of the genetic variation among our modern samples (amova, *p *< .001). This is substantially greater than the ~10% of variance deriving from simple IBD, and supports the hypothesis that the geographical features (major rivers, deserts and mountain chains) define population structure in contemporary wolves across the Northern Hemisphere and therefore constitute obstacles to gene flow (but where the strength of these obstacles may vary).

### 3.2 Bayesian phylogenetic analysis

All ancient sequences included in the study were subjected to stringent quality criteria with respect to coverage and damage patterns. Of the 45 ancient samples, 38 had well‐resolved direct radiocarbon dates. We joined these ancient sequences with 90 modern mitogenome sequences and used beast (Drummond et al., 2012) to estimate a wolf mitochondrial mutation rate. By applying the inferred mutation rate we were able to molecularly date the remaining seven ancient sequences (Materials and Methods). We cross‐validated this approach through a leave‐one‐out analysis (Materials and Methods) using all the directly dated ancient sequences and found a very close fit (*R* ^2^ = 0.86) between the radiocarbon and the estimated molecular dates and no systematic biases in our molecularly estimated dates (Figure S9), meriting the inclusion of these sequences and the inferred dates into the spatially explicit analyses.

**Figure 2:**
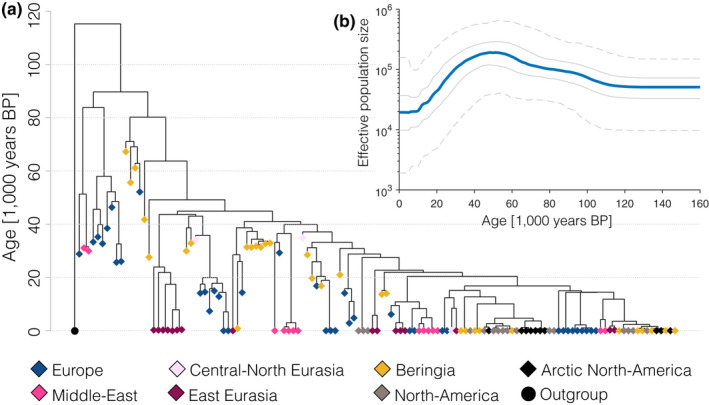
(a) Tip calibrated beast tree of all samples used in the spatial analyses (diamonds), coloured by geographical region. The circle represents an outgroup (modern Indian wolf, not used in the analyses). (b) The effective population size through time from the beast analysis (Bayesian skyline plot). The solid blue line represents the median estimate and the grey lines represent the interquartile range (sold lines) and 95% intervals (dashed lines) [Colour figure can be viewed at wileyonlinelibrary.com]

Our Bayesian phylogenetic analysis suggests that the MRCA of all extant North Eurasian and American wolf mitochondrial sequences dates to ~40,000 years ago, whereas the MRCA for the combined ancient and modern sequences dates to ~90,000 years ago (95% highest posterior density [HPD] interval: 82,000–99,000 years ago) (Figure 2a, see Figures S11 and S12 for node support values and credibility intervals). A divergent clade at the root of this tree consists exclusively of ancient samples from Europe and the Middle East that has not contributed to present‐day mitochondrial diversity in our data (see also Thalmann et al., 2013).

The remainder of the tree consists of a monophyletic clade that is made up of ancient and modern samples from across the Northern Hemisphere that shows a pattern of rapid bifurcations of genetic lineages centred on 25,000 years ago. To further quantify this temporal pattern, we made use of a Bayesian skyline analysis (Figure 2b) that shows a relatively small and stable effective genetic population size between ~20,000 years ago and the present and a decrease in effective population size between ~40,000 and 20,000 years ago. This pattern is consistent with the scenario suggested in whole genome studies (e.g. Fan et al., 2016; Freedman et al., 2014) where wolves had a stable (and probably geographically structured) population across the Northern Hemisphere up to a time point between 20,000 and 30,000 years ago, when the population experienced a bottleneck that severely reduced genetic variation followed by a rapid population expansion.

The samples at the root of this clade are predominantly from Beringia, pointing to a possible expansion out northeast Eurasia or the Americas. However, given the uneven temporal and geographical distribution of our samples, and the stochasticity of a single genetic marker (Nielsen & Beaumont, 2009), it is important to explicitly test the extent to which this pattern can occur by chance under other plausible demographic scenarios.

### 3.3 Spatiotemporal reconstruction of past grey wolf demography

**Figure 3:**
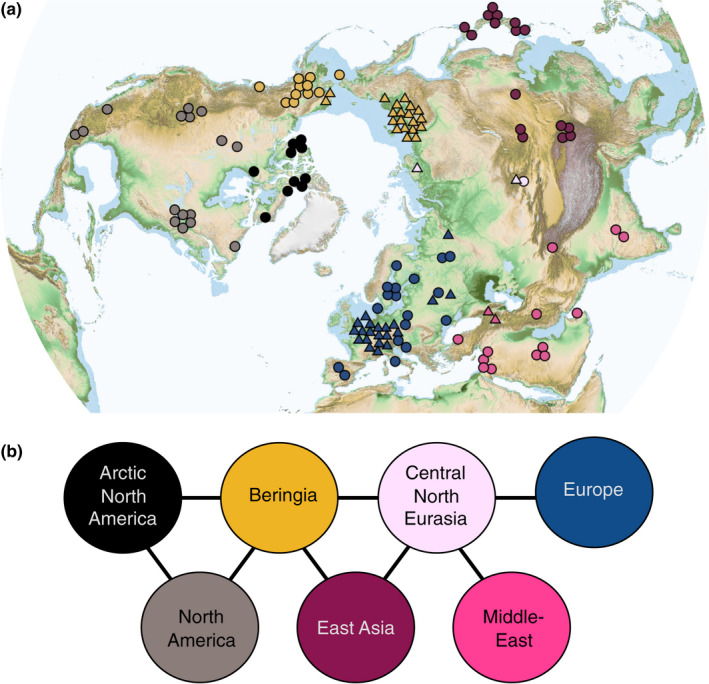
(a) Sample locations and geographical regions, with boundaries indicated by dashed lines. The dark blue indicates sea levels shallow enough to be land during the Last Glacial Maximum (sea depth < 120 m). (b) Model network of populations (“demes”), connected by gene flow, corresponding to the regions in (a)

Having established the phylogenetic relationship between our samples and population structure across the Northern Hemisphere, we tested the ability of different explicit demographic scenarios to explain the observed phylogenetic pattern, while also taking into account the geographical location and age of each sample. To this end, we represented each of the regions in Figure 3(a) as a population in a network of populations connected by gene flow (Figure 3b). We used the coalescent population genetic framework to model genetic evolution in this network, in which each deme constitutes a freely mixing and randomly mating population. The effective population size of demes, as well as movement of individuals between demes, are controlled by parameters covering values that represent different demographic histories.

Using this framework we considered a wide range of different explicit demographic scenarios (illustrated in Figure 3a, see Materials and Methods for details of implementation within the coalescent framework). The first scenario consisted of a constant population size and uniform movement between neighbouring demes. This allowed us to test the null hypothesis that drift within a structured population alone can explain all the patterns observed in the mitochondrial tree. We then considered two additional demographic processes that could explain the observed patterns: (a) a temporal sequence of two population size changes that affected all demes simultaneously (thus allowing for a bottleneck); and (b) an expansion out of one of the seven demes. In the expansion scenarios, the deme of origin had a continuous population through time, while in the remaining demes the indigenous population was sequentially replaced by the expanding population. Scenario 2 was repeated for all seven possible expansion origins, thus allowing us to test continuity as well as replacement hypotheses within each of the seven demes. We considered each demographic event in isolation as well as their combined effect (resulting in a total of 16 scenarios) and used ABC to calculate the likelihood of each scenario and estimate parameter values (see Materials and Methods for details).

**Figure 4:**
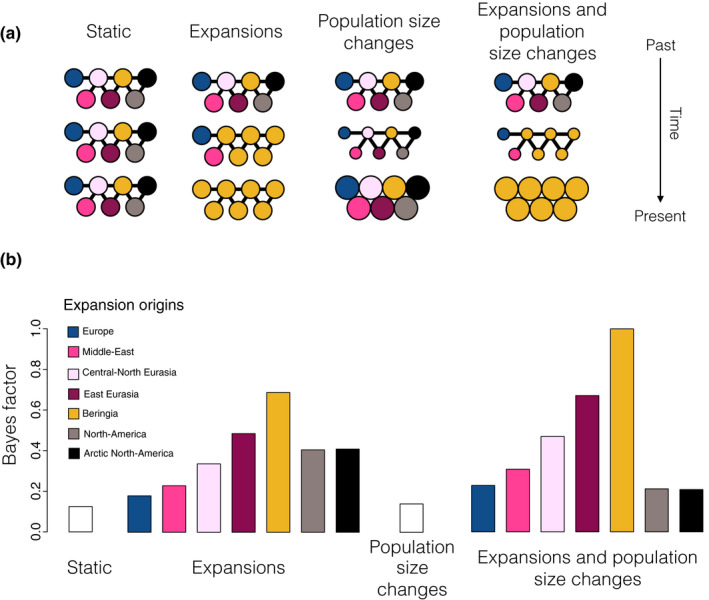
Spatially and temporally explicit analysis. (a) Illustration of the different scenarios, with circles representing one deme each for the seven different geographical regions (see panel b for colour legend and text for full description of the scenarios). Solid lines represent population connectivity. The *static* scenario (far left) shows stable populations through time. The *expansion* scenario (middle left) shows how one deme (here yellow) expands and sequentially replaces the populations in all other demes (from top to bottom). The *population size change* scenario (middle right) illustrates how population size in the demes can change through time (large or small population size shown as large or small circles, respectively). We also show a combined scenario (far right) of both expansion and population size change. (b) Likelihood of each demographic scenario relative to the most likely scenario, shown as Bayes factors, estimated using Approximate Bayesian Computation analyses (see text for details). For expansion scenarios (including the combined expansion and population size changes), we colour code each bar according to the origin of the expansion (see colour legend)

Both the null scenario and the scenario of only population size change in all demes were strongly rejected (BF ≤ 0.1, Figure 4b; Table S6), illustrating the power of combining a large data set of ancient samples with statistical modelling. Scenarios that combined an expansion and replacement with a change in population size (bottleneck) were better supported than the corresponding scenarios (i.e., with the same expansion origin) with constant population size (Figure 4b).

**Figure 5:**
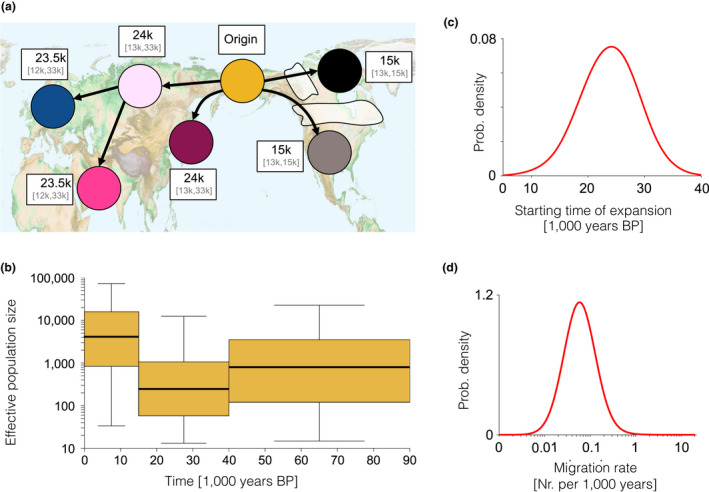
The inferred scenario of wolf demography from the Bayesian analysis using our spatially and temporally explicit model (see Figure 4 and the main text). (a) Geographical representation of the expansion scenario (out of Beringia) with median and 95% confidence interval (CI) for the date of the population replacement in each deme given in white boxes next to each deme. (b) Effective population size (thick line, boxes and whiskers show the median, interquartile range and 95% CI, respectively, for each time period). (c) Posterior distribution of migration rate and (d) starting time of expansion [Colour figure can be viewed at wileyonlinelibrary.com]

The best‐supported scenario (Figure 5) was characterized by the combination of a rapid expansion of wolves out of the Beringian deme ~ 25,000 years ago (95% confidence interval [CI]: 33,000–14,000 years ago) with a population bottleneck between 15,000 and 40,000 years ago, and limited gene flow between neighbouring demes (see Table S7 and Figure S13 for posterior distributions of all model parameters). We also found relatively strong support for a scenario that describes a wolf expansion out of the East Eurasian deme (BF 0.7) with nearly identical parameters to the best‐supported scenario (Table S8 and Figure S14). This can be explained by the geographical proximity of East Eurasian and Beringian demes and the genetic similarity of wolves from these areas.

## 4 DISCUSSION

### 4.1 Geographical origin of the ancestral wolf population

Recent whole‐genome studies (Fan et al., 2016; Freedman et al., 2014; Skoglund et al., 2015) found that modern grey wolves (*Canis lupus*) across Eurasia are descended from a single source population. The results of our analyses combining both ancient and modern grey wolf samples (Figure 1) with a spatially and temporally explicit modelling framework (Figure 4) suggest that this process began ~25,000 (95% CI: 33,000–14,000) years ago when a population of wolves from Beringia (or a Northeast Asian region in close geographical proximity) expanded outwards and replaced indigenous Pleistocene wolf populations across Eurasia (Figure 5). This scenario also provides a mechanism explaining the star‐like topology of modern wolf populations observed in whole genome studies (Fan et al., 2016; Freedman et al., 2014; Skoglund et al., 2015): the expansion was split up by geographical barriers that restricted subsequent gene flow between different branches of the expanding population, which in turn led to the divergence between different subpopulations observed in contemporary grey wolves.

In the Americas, the Beringian expansion was delayed due to the presence of ice sheets extending from Greenland to the northern Pacific Ocean (Figure 5) (Raghavan et al., 2015). A study by Koblmüller et al. (2016) suggested that wolf populations that were extant south of these ice sheets were replaced by Eurasian wolves crossing the Beringian land bridge. Our data and analyses support the replacement of North American wolves (following retreat of the ice sheets around 16,000 years ago), and our more extensive ancient DNA sampling, combined with spatially explicit modelling, has allowed us to narrow down the geographical origin of this expansion to an area between the Lena River in Russia and the Mackenzie River in Canada also known as Beringia (Hopkins, Matthews, & Schweger, 1982). However, due to lack of Pleistocene wolf samples that pre‐date the retreat of the ice sheets in the area, we are currently not able to resolve the detailed history of North American wolves. For example, we cannot reject an alternative scenario where contemporary North American wolves are descendants of a Pleistocene wolf population that was genetically highly similar to the Beringian population but existed south of the ice sheets.

Thus, despite a continuous fossil record through the Late Pleistocene, wolves experienced a complex demographic history involving population bottlenecks and replacements (Figure 5). Our analysis suggests that long‐range migration played an important role in the survival of wolves through the wave of megafaunal extinctions at the end of the last glaciation. These results will enable future studies to examine specific local climatic and ecological factors that enabled the Beringian wolf population to survive and expand across the Northern Hemisphere. Furthermore, as the reconstructions in this study are based solely on a maternally inherited genetic marker, our model was thus only able to address a set of simplified demographic scenarios (continuity everywhere, or continuity in one location followed by a replacement expansion from it). Once whole‐genome data become available, it will probably be possible to detect contributions from potential refugia at the local scale.

### 4.2 Implications for the evolution of grey wolf morphology

Morphological analyses of wolf specimens have noted differences between Late Pleistocene and Holocene wolves: Late Pleistocene specimens have been described as craniodentally more robust than present‐day grey wolves, as well as having specialized adaptations for carcass and bone processing (Baryshnikov, Mol, & Tikhonov, 2009; Kuzmina & Sablin, 1993; Leonard et al., 2007) associated with megafaunal hunting and scavenging (Fox‐Dobbs, Leonard, & Koch, 2008; Germonpré et al., 2017). The early Holocene archaeological record has only yielded a single sample with the Pleistocene wolf morphotype (in Alaska) (Leonard et al., 2007), suggesting that this robust ecomorph had largely disappeared from the Northern Hemisphere by the Pleistocene–Holocene transition. This change in wolf morphology coincides with a shift in wolf isotope composition (Bocherens, 2015), and the disappearance of megafaunal herbivores and other large predators such as cave hyenas and cave lions, suggesting a possible change in the ecological niche of wolves.

To date, it has been unclear whether the morphological change was the result of population replacement (genetic turnover), a plastic response to a dietary shift, or both. Our results suggest that the Pleistocene–Holocene transition was accompanied by a genetic turnover in most of the Northern Hemisphere wolf populations as most indigenous wolf populations experienced a large‐scale replacement resulting in the loss of all native Pleistocene genetic lineages (Figure 5). Similar population dynamics of discontinuity and replacement by conspecifics have been observed in several other large Pleistocene mammals in Europe including cave bears, woolly mammoths (Palkopoulou et al., 2013; Stuart, Kosintsev, Higham, & Lister, 2004), giant deer (Stuart et al., 2004) and even humans (Fu et al., 2016; Posth et al., 2016).

The geographical exception to this pattern of widespread replacement is Beringia, where we infer demographic continuity between Late Pleistocene and Holocene wolf populations (Figure 5). This finding is at odds with a previous suggestion of genetic turnover in Beringia (Leonard et al., 2007), probably as the result of differences in both the amount of data available and the analytical methodology used. Leonard et al. (2007) used a short (427 bases long) segment of the mitochondrial control region and employed a descriptive phylogeographical approach, whereas our conclusions are based on an expanded data set in terms of both sequence length, sample number, and geographical and temporal range (Figure 1) and formal hypothesis testing within a Bayesian framework (Figures 4 and 5).

As a consequence, the morphological and dietary shift observed in Beringian wolves between the Late Pleistocene and Holocene (Leonard et al., 2007) cannot be explained by population turnover, but instead requires an alternative explanation such as adaptation or plastic responses to the substantial environmental and ecological changes that took place during this period. Indeed, grey wolves are a highly adaptable species. Studies of modern grey wolves have found that differences in habitat, specifically precipitation, temperature, vegetation and prey specialization, can strongly affect their craniodental morphology (Flower & Schreve, 2014; Geffen et al., 2004; Leonard, 2015; O'Keefe, Meachen, Fet, & Brannick, 2013; Pilot et al., 2006).

The specific causal factors for the replacement of indigenous Eurasian wolves during the LGM by their Beringian conspecifics (and American wolves following the disappearance of the Cordilleran and Laurentide ice sheets) are beyond the scope of this study. However, one possible explanation may be related to the relatively stable climate of Beringia compared to the substantial climatic fluctuations that impacted the rest of Eurasia and Northern America during the Late Pleistocene (Clark et al., 2012). These fluctuations have been associated with dramatic changes in food webs, leading to the loss of most of the large Pleistocene predators in the region (Bocherens, 2015; Hofreiter & Stewart, 2009; Lister & Stuart, 2008; Lorenzen et al., 2011). In addition, the hunting of large Pleistocene predators by late Palaeolithic people (e.g. Cueto, Camarós, Castaños, Ontañón, & Arias, 2016; Germonpré & Hämäläinen, 2007; Münzel & Conard, 2004) may have also negatively impacted large carnivore populations (Fan et al., 2016). An interdisciplinary approach involving morphological, isotopic as well as genetic data is necessary to better understand the relationship between wolf population dynamics and dietary adaptations in the Late Pleistocene and early Holocene period.

### 4.3 Implications for the study of wolf domestication

Lastly, the complex demographic history of Eurasian grey wolves reported here (Figure 5) also has significant implications for identifying the geographical origin(s) of wolf domestication and the subsequent spread of dogs. For example, our limited understanding of the underlying wolf population structure may explain why previous studies have produced conflicting geographical and temporal scenarios. Numerous previous studies have focused on the patterns of genetic variation in modern domestic dogs, but have failed to consider potential genetic variation present in Late Pleistocene wolf populations, thereby implicitly assuming a homogeneous wolf population source. As a result, both the domestication and the subsequent human‐mediated movements of dogs were the only processes considered to have affected the observed genetic patterns in dog populations. However, both domestication from and admixture with a structured wolf population will have consequences for patterns of genetic variation within dogs. In light of the complex demographic history of wolves (and the resulting population genetic structure) reconstructed by our analysis, several of the geographical patterns of haplotype distribution observed in previous studies, including differences in levels of diversity found within local dog populations (Wang et al., 2016), and the deep phylogenetic split between Eastern and Western Eurasian dogs (Frantz et al., 2016), could have resulted from known admixture between domestic dogs and grey wolves (Fan et al., 2016; Freedman et al., 2014; Godinho et al., 2011; Verardi, Lucchini, & Randi, 2006). Future analyses should therefore explicitly include the demographic history of wolves and demonstrate that the patterns of variation observed within dogs fall outside expectations that take admixture with geographically structured wolf populations into account.

## AUTHOR CONTRIBUTIONS

L.L., O.T., M.T.P.G., J.K., G.L., A.E. and A.M. designed the research; O.T., M‐H.S.S., V.J.S., K.E.W., M.S.V., I.K.C.L., N.W. and G.S. performed ancient DNA laboratory work with input from J.K., M.T.P.G., H.S., K‐H.H., and R.S.M.; M‐H.S.S. performed modern DNA laboratory work with input from M.T.P.G; O.T., J.A.S.C. and L.L. performed bioinformatic analyses; L.L., A.E. and A.M. designed the population genetic analyses; L.L. performed phylogenetic analyses; A.E. implemented the spatial analyses framework; L.L and A.E. performed spatial analyses; M.G., J.B., V.V.P., E.Y.P., P.A.N., S.E.F., J.E‐L., A.W.K., B.G., H.N., H‐P.U. and M.L‐G. provided samples; V.V.P., M.G., M.L‐G., H.B., H.N., A.W.K., E.Y.P. and P.A.N. provided context for archaeological samples; A.P., M.G., H.B. and K.D. helped setting the results of genetic analyses into an archaeological context; A.M., M.T.P.G., A.J.H., G.L., J.K., E.W. and K.D. secured funding for the project; L.L., O.T. and A.E. wrote the initial draft of the manuscript with input from A.M.; L.L., O.T. and A.E wrote the manuscript and the supplementary information with input from A.P., M.G., H.B., M‐H.S.S., M.T.P.G., K.E.W., A.M., G.L and K.D.; V.J.S., L.F., A.W.K., K‐H.H., A.J.H., R.S.M., H.S., G.S., V.V.P., E.Y.P., P.A.N. and J.E‐L. provided comments on the manuscript and/or on the supplementary information.

## Data Availability

The newly assembled mitochondrial genomes are available from GenBank (accession numbers MK936995–MK937053 [ancient] and MN071185‐MN071206 [modern]). The raw sequencing reads used for generating novel ancient mitochondrial genomes can be retrieved from the European Nucleotide Archive under study number PRJEB32023. The code for population genetic simulations of all tested scenarios and scripts for preliminary and output analyses are available on the GitHub repository at https://github.com/LiisaLoog/pleistocene-wolves.
