# Supplementary material for: Ancient DNA suggests modern wolves trace their origin to a Late Pleistocene expansion from Beringia
Source: Mol Ecol. 2020 Jan 2;29(9):1596–610. doi: 10.1111/mec.15329 (PMC7317801; doi:10.1111/mec.15329)
Supplement: Supplementary file 2 [file MEC-29-1596-s002.pdf]

MOLECULAR ECOLOGY

Supplemental Information for:

Ancient DNA suggests modern wolves trace their origin to a late Pleistocene expansion from Beringia

Liisa Loog[1,2,3]*, Olaf Thalmann[4†], Mikkel-Holger S. Sinding[5,6,7†], Verena J. Schuenemann[8,9,10†], Angela Perri[11], Mietje Germonpré[12], Herve Bocherens[9,13], Kelsey E. Witt[14], Jose A. Samaniego Castruita[5], Marcela S. Velasco[5], Inge K. C. Lundstrøm[5], Nathan Wales[5], Gontran Sonet[15], Laurent Frantz[2], Hannes Schroeder[5,16], Jane Budd[17], Elodie-Laure Jimenez [12], Sergey Fedorov[18], Boris Gasparyan[19], Andrew W. Kandel[20], Martina Lázničková-Galetová[21,22,23], Hannes Napierala[24], Hans-Peter Uerpmann[8], Pavel A. Nikolskiy[25,26], Elena Y. Pavlova[27,26], Vladimir V. Pitulko[26], Karl-Heinz Herzig[4,28], Ripan S. Malhi[29], Eske Willerslev[2,5,30], Anders J. Hansen[5,7], Keith Dobney[31,32,33], M. Thomas P. Gilbert[5,34], Johannes Krause[8,35], Greger Larson[1]*, Anders Eriksson[36,2]*, Andrea Manica[2]*

MOLECULAR ECOLOGY

Table of Contents:

S1 – ARCHAEOLOGICAL BACKGROUND AND SAMPLE INFORMATION 4

1.1 PALEONTOLOGICAL HISTORY OF GREY WOLVES 4
1.2 ARCHAEOLOGICAL SITE DESCRIPTIONS 8
ARMENIA 8
BELGIUM 8
CZECH REPUBLIC 10
SWITZERLAND 10
RUSSIA 11

S2 – DNA EXTRACTION, SEQUENCING AND BIOINFORMATICS. 21

2.1 DNA EXTRACTIONS 21
2.2 LIBRARY PREPARATION 22
2.3 SEQUENCE GENERATION 24
2.4 RAW SEQUENCE DATA PROCESSING 25
2.5. MOLECULAR CHARACTERISTICS OF NEWLY GENERATED, ANCIENT SEQUENCES 26
Table S2 27
Figure S1 29
Figure S2 30

S3 – DATA ANALYSES & RESULTS 33

3.1 BEAST ANALYSES & RESULTS 33
PARTITION FINDER 33
Table S3 33
MUTATION RATE CALCULATION 33
INPUT FILE SETTINGS 34
PRIORS 34
MCMC CHAIN 35
BEAST RESULTS 35
Table S4 35
Table S5 35

MOLECULAR ECOLOGY

3.2 SPATIAL MODELLING RESULTS **36**
Table S6 36
Table S7 37
Table S8 37
3.3 SUPPLEMENTARY FIGURES **39**
Figure S3 39
Figure S4 40
Figure S5 41
Figure S6 42
Figure S7 43
Figure S8 44
Figure S9 45
Figure S10 46
Figure S11 47
Figure S12 48
Figure S13 49
Figure S14 50

MOLECULAR ECOLOGY

S1 – Archaeological Background and Sample Information

1.1 Paleontological history of grey wolves

Grey wolves (*Canis lupus*) are a highly adaptable species, able to live in a range of environments and with a wide natural distribution. Studies of modern grey wolves have found distinct subpopulations living in close proximity (Musani et al., 2007; Schweizer et al., 2016). This variation is closely linked to differences in habitat - specifically precipitation, temperature, vegetation, and prey specialization, which particularly affect cranio-dental plasticity (Geffen et al., 2004; Pilot et al., 2006; Flower and Schreve, 2014; Leonard, 2015).

The earliest evidence for *Canis lupus* comes from the sites of Cripple Creek Sump (Alaska, United States) and Old Crow (Yukon, Canada; Tedford et al. 2009), indicating Eastern Beringia as the likely center of origin. The age of this material may be up to 1.0 Ma, though the geological attribution and dating is controversial (Repenning 1992, Tedford et al. 2009, Westgate et al. 2013). In Eurasia, *Canis lupus* appears nearly simultaneously during the late Middle Pleistocene, including in Siberia (500-300 ka BP; Sotnikova and Rook 2010), France (400-350 ka BP; Bonifay, 1971, Brugal and Boudadi-Maligne, 2011), and Italy (340-320 ka BP; Anzidei et al. 2012), probably representing the origin of true modern grey wolves (Sardella et al. 2014). By the end of the Middle Pleistocene, grey wolves are found across all of Eurasia (e.g. Kahlke, 1994; Boeskorov and Baryshnikov, 2013).

Late Pleistocene seems to harbor considerable morphological diversity among wolves. However, the Pleistocene wolf morphotypes have been described as cranio-dentally more robust than the present day grey wolves and as having specialized adaptations (e.g., shortened rostrum, pronounced development of the temporalis muscle, robust premolars) for carcass and bone processing (Kuzmina and Sablin 1995, Leonard et al. 2007, Baryshnikov et al. 2009) associated with megafaunal hunting and scavenging.

First described by Olsen (1985) as “short-faced wolves”, a Late Pleistocene hypercarnivorous grey wolf morph with a, broad snout, robust mandible, and large carnassials used for targeting Pleistocene megafauna and scavenging carcasses has been more recently referred to as the Beringian wolf (Leonard et al., 2007; Baryshnikov et al., 2009; Boeskorov and Baryshnikov, 2013; Meachen et al., 2016, Germonpré et al., 2017). The ecomorph is known from just two sites – the eastern Beringian type site in Fairbanks, Alaska (specimens dated to before 50-12.5 ka; Olsen 1985, Leonard 2007) and Natural Trap Cave, Wyoming in the northern continental United States (specimens dated to 25.8-14.3 ka; Kohn and McKay 2012, Meachen et al., 2016). However, similar robust ecomorphs have been found from western Beringia. Baryshnikov et al. (2009) described a small, but robust wolf also specialized for megafaunal hunting and scavenging from Lake Taimyr, Siberia (dated to c. 19.3 ka; MacPhee et al., 2002). They associated this Lake Taimyr wolf with a similar morphological wolf subspecies from Late Pleistocene Europe, the cave (or “Ice Age”) wolf (*Canis lupus spelaeus/Canis lupus brevis*), also a specialized Pleistocene ecomorph. Like the Beringian and Lake Taimyr wolves, the cave wolf was smaller-bodied, but

robust cranio-dentally for megafaunal hunting and scavenging (Stiner 2004, Baryshnikov et al., 2009, Diedrich 2013). Though there are clear affinities between these Late Pleistocene morphs, there is very little associated data and more work is needed to better understand any relationships between them. Though a single Pleistocene "Beringian" wolf morphotype has been documented from early Holocene Alaska, most specimens date to the Late Pleistocene, suggesting this Late Pleistocene ecomorph had disappeared by the Pleistocene-Holocene transition, coinciding with the disappearance of many megafaunal herbivores.

The east Beringian environment during the Late Pleistocene was a cold glacial steppe populated by megafauna that included mammoth, bison, horse and muskox. Stable isotope analysis of Beringian wolves confirms that they had a diet consisting of a mix of megafaunal prey species (Leonard et al., 2007), though mammoth may have been rare in their diets (Fox-Dobbs et al., 2008). In fact, the diet of Beringian wolves may have differed depending on the temporal and climatic conditions in which they lived. In the pre-glacial period (up to c. 22 ka), some specialized in forest herbivores (woodland muskox and deer), while others were generalists. During the Last Glacial Maximum (LGM: c. 21-17 ka; Tamm et al., 2007; Pilot et al., 2010) some wolves appear to have been mammoth specialists – though it is unclear if they were hunters or scavengers of this prey – and only the early Holocene wolf had a diet of forest cervids (Fox-Dobbs et al., 2008). It has been found that Pre-LGM European wolves have also had a diet constant with high proportion of mammoth (Bocherens et al., 2015). The diet of the western Beringian Lake Taimyr wolf has yet to be analysed.

Bibliography:

AnzideI, A.P., Bulgarelli, G.M., Catalano, P., Cerilli, E., Gallotti, R., Lemorini, C., Milli, S., Palombo, M.R., Pantano, W. and Santucci, E., 2012. Ongoing research at the late Middle Pleistocene site of La Polledrara di Cecanibbio (central Italy), with emphasis on human–elephant relationships. *Quaternary International*, 255, pp.171-187.

Baryshnikov, G.F., Mol, D., Tikhonov, A.N., 2009. Finding of the Late Pleistocene carnivores in Taimyr peninsula (Russia, Siberia), with paleoecological context. Russian Journal of Theriology 8, 107-113.

Bocherens, H., Drucker, D.G., Germonpré, M., Lázničková-Galetová, M., Naito, Y.I., Wissing, C., Brůžek, J., Oliva, M., 2015. Reconstruction of the Gravettian food-web at Předmostí I using multi-isotopic tracking (^{13}C, ^{15}N, ^{34}S) of bone collagen. Quaternary International 359-360, 211-228.

Boeskorov, G.G.,Baryshnikov, G.F., 2013. Late Quaternary Carnivora of Yakutia. Saint-Petersburg: Nauka. 199 p. [in Russian]

Bonifay, M.-F., 1971. Carnivores quaternaires du sud-est de la France. Mémoires du Muséum National d'Histoire Naturelle, Série C, 21, 49-377.

Boeskorov, G.G., Baryshnikov, G.F., 2013. Late Quaternary Carnivora of Yakutia.Nauka, Saint-Petersburg.

Brugal, J.-P., Boudadi-Maligne, M., 2011.Quaternary small to large canids in Europe: Taxonomic statusand biochronological contribution. Quaternary International 243, 171-182.

Diedrich, C.G., 2013. Extinctions of Late Ice Age cave bears as a result of climate/habitat change and large carnivore lion/hyena/wolf predation stress in Europe. *ISRN Zoology*, 2013, pp. 1-25.

Flower, L.O., Schreve, D.C., 2014. An investigation of palaeodietary variability in European Pleistocene canids. Quat. Sci. Rev. 96, 188-203.

Fox-Dobbs, K., Leonard, J.A., Koch, P.L., 2008. Pleistocene megafauna from eastern Beringia: Paleoecological and paleoenvironmental interpretations of stable carbon and nitrogen isotope and radiocarbon records. Palaeogeography, Palaeoclimatology, Palaeoecology 261, 30-46.

Geffen, E.L.I., Anderson, M.J., Wayne, R.K., 2004. Climate and habitat barriers to dispersal in the highly mobile grey wolf. Mol. Eco. 13 (8), 2481e2490.

Germonpré, M., Fedorov, S., Danilov, P., Galeta, P., Jimenez, E.-L., Sablin, M., Losey, R.J., 2017. Palaeolithic and prehistoric dogs and Pleistocene wolves from Yakutia: Identification of isolated skulls. J. Archaeol. Sc 78, 1-19.

Kahlke, R.D., 1994. Die Entstehungs-, Entwicklungs- und Verbreitungsgeschichte des oberpleistozänen Mammuthus-Coelodonta-Faunenkomplexes in Eurasien (Grosssäuger). Abhandlungen der Senckenbergischen Naturforschenden Gesellschaft 546, 1-164.

Kohn, M.J., McKay, M.P., 2012. Paleoecology of late Pleistocene–Holocene faunas of eastern and central Wyoming, USA, with implications for LGM climate models. Palaeogeography, Palaeoclimatology, Palaeoecology 326, 42-53.

Kuzmina, I.E., Sablin, M.V., 1993. Pozdnepleistotsenovyi pesets verhnei Desny. In: Baryshnikov, G.F., Kuzmina, I.E. (Eds.), Materiali po mezozoickoi i kainozoickoi istorii nazemnykh pozvonochnykh. Trudy Zoologicheskogo Instituta RAN 249, pp. 93–104 [in Russian with English summary].

Leonard, J.A., Vilà, C., Fox-Dobbs, K., Koch, P.L., Wayne, R.K., Van Valkenburgh, B., 2007. Megafaunal extinctions and the disappearance of a specialized wolf ecomorph. Current Biology 17, 1146-1150.

Leonard, J.A., 2015. Ecology drives evolution in grey wolves. *Evolutionary Ecology Research*, 16(6), pp.461-473.

MacPhee, R.D., Tikhonov, A.N., Mol, D., De Marliave, C., Van Der Plicht, H., Greenwood, A.D., Flemming, C. Agenbroad, L., 2002. Radiocarbon chronologies and extinction dynamics of the Late Quaternary mammalian megafauna of the Taimyr Peninsula, Russian Federation. *Journal of Archaeological Science*, 29(9), pp.1017-1042.

Meachen, J.A., Brannick, A.L., Fry, T.J., 2016. Extinct Beringian wolf morphotype found in the continental U.S. has implications for wolf migration and evolution. Ecology and Evolution 6, 3430-3438.

Musiani, M., Leonard, J.A., Cluff, H., Gates, C.C., Mariani, S., Paquet, P.C., Vilà, C.,

Wayne, R.K., 2007. Differentiation of tundra/taiga and boreal coniferous forest wolves: genetics, coat colour and association with migratory caribou. Mol. Ecol. 16 (19), 4149-4170.

Olsen, S.J., 1985. Origins of the Domestic Dog: The Fossil Record. University of Arizona Press, Tucson.

Pilot, M., Branicki, W., Je¸drzejewski, W., Goszczy_nski, J., Je¸drzejewska, B., Dykyy, I., Shkvyrya, M., Tsingarska, E., 2010. Phylogeographic history of grey wolves in Europe. BMC Evol. Biol. 10, 104.

Repenning, C.A., 1992. Allophaiomys and the age of the Olyor Suite, Krestovka sections, Yakutia. U.S. Geological Survey Bulletin 2037: 1–98.

Sardella, R., Bertè, D., Iurino, D.A., Cherin, M. and Tagliacozzo, A., 2014. The wolf from Grotta Romanelli (Apulia, Italy) and its implications in the evolutionary history of *Canis lupus* in the Late Pleistocene of Southern Italy. *Quaternary International*, 328, pp.179-195.

Schweizer, R.M., vonHoldt, B.M., Harrigan, R., Knowles, J.C., Musiani, M., Coltman, D., Novembre, J., Wayne, R.K., 2016. Genetic subdivision and candidate genes under selection in North American gray wolves. Molecular Ecology 25, 380-402.

Sotnikova, M., Rook, L., 2010. Dispersal of the Canini (Mammalia, Canidae: Caninae) across Eurasia during the Late Miocene to Early Pleistocene. Quaternary International, 212 (2). 86-97.

Stiner, M.C., 2004. Comparative ecology and taphonomy of spotted hyenas, humans, and wolves in Pleistocene Italy. *Revue de Paléobiologie*, 23(2), pp.771-785.

Tamm, E., Kivisild, T., Reidla, M., Metspalu, M., Smith, D.G., Mulligan, C.J., Bravi, C.M., Rickards, O., Martinez-Labarga, C., Khusnutdinova, E.K., Fedorova, S.A., Golubenko, M.V., Stepanov, V.A., Gubina, M.A., Zhadanov, S.I., Ossipova, L.P., Damba, L., Voevoda, M.I., Dipierri, J.E., Villems, R., Malhi, R.S., 2007. Beringian standstill and spread of Native American founders. PLoS One 9, 8291–8296.

Tedford, R.H., Wang, X., Taylor, B.E., 2009. Phylogenetic systematic of the North American fossil Caninae (Carnivora, Canidae). Bulletin of the American Museum of Natural History, 325.

Westgate, J.A., Pearce, G.W., Preece, S. J., Schweger, C.E., Morlan, R.E., Pearce, N.J.G., Perkins, T.W., 2013. Tephrochronology, magnetostratigraphy and mammalian faunas of Middle and Early Pleistocene sediments at two sites on the Old Crow River, northern Yukon Territory, Canada. Quaternary Research 79 (1), 75-85

1.2 Archaeological site descriptions

Armenia

Aghitu (TU9, TU10)

Aghitu-3 is a shallow cave at the base of a 126-111 ka basalt flow, located in a paleomeander of the Vorotan River, a tributary of the Araxes River, which flows into the Caspian Sea. Between 2009 and 2015, A.W. Kandel and B. Gasparyan excavated this 5 m-thick, Upper Paleolithic sequence dated between 40-24,000 y cal BP. Faunal assemblages from five cultural horizons (AH VII-III) contain mainly wild goat, wild sheep and horse, with other species being less frequent (Kandel et al., 2014). Two mitochondrial genomes were newly sequenced and analyzed in this study. The first sample is from a well-preserved, complete cranium including both mandibles (TU10) of a large canid dated to 30,000 y cal BP found at the interface of layers AH Ve and VI; the specimen has cut marks, likely made by stone tools, as well as bite marks, likely made by a canid-sized carnivore. The second sample is from a well-preserved proximal left radius (TU9) of a large canid dated to 31,100 y cal BP found near the top of layer AH VI.

Belgium

Goyet, the third cave (TH2, TH3, TU6)

The third cave of Goyet is located in the Belgian Mosan basin. This cave was first excavated in 1868 by the geologist Edouard Dupont (1872). He described five successive bone horizons. The material from the Dupont excavations is housed at the Royal Belgian Institute of Natural Sciences (RBINS) and consists of at least 30,000 bone fragments (Rougier et al., 2016b). Mitochondrial genomes from two canid elements from the Goyet third cave were analyzed in this study: a mandible (TH3) and an upper carnassial (P4) (TU6), which was newly sequenced and analyzed in this study. According to Dupont's unpublished notes, the lower jaw, with an age of 28,800 y cal BP, was found in Bone Horizon 4 in a side gallery adjacent to the entrance of the cave, together with remains from mammoth, lynx, red deer and large canids. The upper carnassial was excavated from Bone Horizon 2.

Pont-à-Lesse, Trou Magrite (TU1, TU2, TU3)

This large cave lies about 3 km upstream of the confluence of the Lesse River with the Meuse River. Although E. Dupont (1867), who started the excavations in 1867, distinguished four bone-bearing layers, the exact provenance of the bones has not been noted, and their stratigraphic position is unfortunately not available. At the RBINS, the Dupont collection of the mammal remains from the Trou Magrite cave amounts to about 50,000 specimens (Jimenez et al, 2016, Smolderen, 2016). The mammal assemblages are primarily composed of remains of horse, reindeer and woolly rhinoceros, some of which show evidence of gnawing by cave hyenas (Jimenez, 2016). The wolves form the most frequent carnivore group, although, few cave bear, cave hyena and cave lion bones have been discovered (Jimenez, 2016). According to Dupont (unpublished notes), the remains of wolves were found together with the consumption refuse and the wolves were likely eaten by the prehistoric people. However, no clear

anthropogenic traces could be discerned on the wolf bones that are all broken. Mitochondrial genomes from three large canid specimens have been newly sequenced and analyzed in this study: two maxilla fragments (TU1 and TU2) with ages of resp. 35,200 y cal BP and 33,300 y cal BP, and a distal tibia fragment (TU3) with an age of 32,700 y cal BP.

Walzin, Trou de l'Ours (TU5)

Trou de l'Ours, a long and small cave that is a part of the Walzin cave complex, is located on the left bank of the Lesse River, a tributary of the Meuse River. E. Dupont started the excavations here in 1866. He noted that the cave was used by badgers. The assemblages from the three bone-bearing layers contain cave bear, elk, wild boar and horse, among other species (Dupont, 1867; Dupont, unpublished notes; Ehrenberg, 1966). A mitochondrial genome from an upper carnassial (TU5) from a large canid, dated to 12,800 y cal BP has been newly sequenced and analyzed in this study.

Furfooz, Trou des Nutons (TH1)

The Trou des Nutons cave is situated in a limestone cliff on the right bank of the Lesse River. This site was also excavated by E. Dupont in the 1860s. The main bone horizon yielded Magdalenian artefacts and the Pleistocene mammal assemblage includes horse and reindeer. However, this horizon also contains younger material, such as remains from sheep, goat, pig, badger, as well as older Pleistocene bones (Charles, 1998). A partly associated skeleton of a large canid excavated by E. Dupont was identified as wolf (Dupont unpublished notes, Germonpré et al., 2009). Interestingly, the right humerus displays cut marks. The skull has an age of 26,000 y cal BP. Previously published mitochondrial genome from this specimen (TH1) is analyzed in this study.

Furfooz, Trou du Frontal (TU4)

This small cave is located on the right bank of the Lesse River near the Trou des Nutons cave. Excavations by E. Dupont started here in 1864. The mammal assemblage contains a mixture of remains dating from the Late Glacial and the Postglacial. A cut-marked horse bone has an age of 15,300 y cal BP (Charles, 1998). A mitochondrial genome from a radius of a large canid (TU4) dated to 6,200 y cal BP was newly sequenced and analyzed in this study.

Furfooz, Trou de Praules (TU8)

This small cave is located on the left bank of the Lesse River 30 m above the river bed and was excavated by E. Dupont in 1866. The mammal assemblage includes remains from reindeer, red deer, horse and brown bear (Dupont, unpublished notes). Lithic artefacts were also discovered (Dupont, 1866). A

mitochondrial genome from a canid mandible (TU8) dated to 7,400 y cal BP was newly sequenced and analyzed in this study.

Hastière, Caverne Marie-Jeanne (TU7)

The Caverne Marie-Jeanne in the Belgian Mosan basin is a cave site located on the right bank of the Féron, a small tributary of the Meuse River near Hastière-Lavaux. Excavations at this site were initiated by M. Glibert of the RBINS in 1943. The cave deposits are composed of clay, silt, and sand that had been washed into the cave along joints. Several bone-bearing layers were described (de Heinzelin, 1980). AMS dates of level 2 suggests a mixture of material dating from the Late Glacial (14,400 y cal BP) and the LGM (25,000 y cal BP), AMS dates from layers 4, 5 and 6 are at least resp. 46,800 y cal BP, 44,500 y cal BP and > 43,000 y cal BP years old (Brace et al., 2012). The mammal assemblages from layers 3 and 4 are dominated by remains from cave hyena, foxes, horse, woolly rhinoceros and large bovids (Jimenez, 2016). The microfauna includes lemmings, voles and mice (Gautier, 1980). A mitochondrial genome from a canid mandible (TU7) from this cave was newly sequenced and analyzed in this study. No information on its stratigraphic position is available but the specimen has been dated to 46,300 y cal BP.

Czech Republic

Předmostí (TU14, TU15, TU16, TU17)

Předmostí is an open air site located in the Moravian Corridor, Czech Republic. The first organized excavations started in the 1880s (Absolon and Klíma, 1977; Oliva, 1997, Svoboda 2008). The mammal assemblage is dominated by mammoths that most likely were hunted and eaten by the Gravettian inhabitants of the site (Oliva, 1997; Bocherens et al., 2015). Large canids are the second most abundant group, with a minimum number of individuals of 120 individuals based on the mandibles (Germonpré et al., 2015). Two morphotypes have been described among the large canid material: Palaeolithic dogs and Pleistocene wolves (Germonpré et al., 2012; Germonpré et al., 2015). Mitochondrial genomes from four canid jaw bones were newly sequenced and analyzed in this study. TU14 is directly dated to 2900 y cal BP, the other three specimens date back to Pleistocene based on dated context. The three Pleistocene specimens (TU15, TU16, TU17) have also been analyzed for the $\delta^{13}C$ and $\delta^{15}N$ values of their bone collagen (Bocherens et al., 2015).

Switzerland

Kesserloch (TU11, TU12, TU13 & TH7, TH11, TH15)

Kesserloch is a cave site located near Thayngen in the Swiss canton of Schaffhausen, in the northernmost part of Switzerland. It was excavated between 1874 and 1903 in several campaigns by three different excavators (published in Merk 1875, Nüesch 1904, Heierli 1907). It lies within the maximum extension of the late glacial alpine ice sheet. Among the rich faunal assemblage that was reviewed in 2007, reindeer, horse and snow hare are the most frequent species both by number and by bone weight (Napierala

2008). Among the carnivores, the canids are the most numerous, dated from around 17,500 to 14,300 y cal BP (Merck, 1876; Höneisen, 1986; Napierala, 2008; Napierala and Uerpmann, 2012). Isotopic investigations of the trophic structure of this fauna revealed that the large canids were predators of large ungulates from the site (Bocherens et al., 2011; Bocherens, 2015). Mitochondrial genomes from three canid specimens have been newly sequenced and analyzed for this study: a maxilla (TU11) (described in Napierala & Uerpmann 2010), another maxilla (TU12) very similar in preservation to TU11, a right mandibular fragment from a large canid (TU13), all directly dated to c. 14,100 y cal BP. Three additional previously published canid sequences (Thalmann et al., 2013) from Kesserloch cave have been analyzed in this study (TH7, TH11, TH15).

Russia

Badyarikha River site (CGG34)

The Badyarikha River site **in northeastern Arctic Siberia** is located on the left bank of the Badyarikha River (67°54'49"N, 146°30'56"E"), a tributary of the **Indighirka** River (Germonpré et al., 2017). The total height of the permafrost cliff is about 20 m. A mitochondrial genome from an isolated canid skull (CGG34), collected 6 m above the waterline and dated to c. 29,900 y cal BP has been newly sequenced for this study. There is no archaeological context associated with this find.

Tirekhtyakh River site (CGG32)

The Tirekhtyakh River site is situated 5 km from the junction of the Tirekhtyakh tributary with the **Indighirka** River (68°53'39"N, 147°12'45"E) in **northeastern Arctic Siberia** (Germonpré et al., 2017). The river terrace here is 8 m high. An isolated canid skull was discovered 2 m below the surface. A mitochondrial genome from this specimen (CGG32) has been newly sequenced and analyzed in this study. Direct radiocarbon dating suggests that this specimen is more than 50,000 years old. There is no archaeological context associated with this find.

Ulakhan Sular site (CGG33)

The Ulakhan Sular site in **northeastern Arctic Siberia** (67°41'40"N, 135°44'24"E) is a 65 m high and 1.2 km long bluff located on the right bank of the Adycha River, a tributary of the Yana River (Germonpré et al., 2017). According to Lee et al. (2015), four main stratigraphic units are present. The lowest unit (layer 1) consists of gravel and pebbles in a sandy matrix. The fauna includes remains from *Archidiskodon* sp., *Equus verae* and *Praealces* sp. and dates from the Early Pleistocene. The faunal assemblage of the next unit (layer 2) consists of bones from *Panthera* sp., *E.nordostensis, Cervalces latifrons* (Lazarev, 2002) and *Canis* cf. *variabilis* (Lee et al., 2015). The third stratigraphic unit (layer 3) is a loamy sand deposit and its fauna includes *Bison priscus crassicornis* remains. The sequence is topped by layer 4 that consists of silty sands with a peat layer. Here, the faunal assemblage contains typical mammoth species such as woolly

mammoth, woolly rhinoceros, horse, red deer, reindeer, bison, muskox, bear, lion and wolf (Lazarev, 2002). A mitochondrial genome from a canid skull, with unknown stratigraphic position (CGG33), dated to 16,900 y cal BP has been newly sequenced and analyzed in this study. There is no archaeological context associated with this find.

Malyi Lyakhovsky Island (CGG31)

Malyi Lyakhovsky Island is one the New Siberian Islands that separate Laptev and East Siberian Sea in The Siberian Arctic. A canid skull was collected on the south coast of this island near a river mouth at the base of a 20 m high permafrost cliff (Germonpré et al., 2017). A mitochondrial genome from this specimen (CGG31), dated to 800 y cal BP has been newly sequenced for this study. There is no archaeological context associated with this find.

Duvannyi Yar, Kolyma River downstream (CGG12, CGG13)

Duvanny Yar is situated on the right bank of the lower Kolyma River in northeastern Arctic Siberia at 68°37'N; 159°06'E (Kaplina et al., 1978; Sher et al., 1979). This 12-km-long outcrop of polifacial permafrost sediments reaches up to 53 m height. Giterman et al. (1982) have distinguished four stratigraphic units within the sequence. Unit One which is lacustrine silts filling in the ice-wedge casts, is exposed at the water level. It is suggested to be last interglacial. Sediments rich with woody plant and peaty organics of the Unit Two are middle Late Pleistocene, while Unit Three is described as loess-like ice and organic-rich permafrost deposits. These compose the most of the sequence. Numerous remains of mammals and plant remains have been found from this site (Yashina et al., 2012). Vasil'chuk et al. (2001) as well as Zanina et al. (2011) suggested that the Unit Three deposits accumulated between >45,000 and 13,500 ^{14}C BP. The uppermost Unit Four is the Holocene cover on the top of the sequence. *Canis lupus* remains from this site, are associated with the lowermost portion of the Unit Three. The age of these fossils is c. 45,000 ^{14}C BP or older. A mitochondrial genome from two canid mandibls from this site (GGG12 and CGG13), both radiocarbon dated to be older than 45,000 y cal BP (Lee et al., 2015) have been newly sequenced and analyzed in this study. This sample was collected by Pavel Nikolskiy in 2005 (GIN RAS accessing number 1131-1). There is no archaeological context associated with these finds.

Yana site

Yana RHS is a complex of geoarchaeological objects that is located in the lower part of the Yana River in northeastern Arctic Siberia. Systematic interdiciplinary investigation of the Yana site complex is led by Pitulko since 2001. The site structure includes several separate localities (at least seven of them) discovered within the body of a river terrace (Pitulko et al., 2004; Pitulko et al., 2013; Pitulko and Pavlova, 2016). Different localities represent separate but roughly contemporaneous archaeological sites. Samples

analyzed in this study come from four different localities (YMAM, Yana/SP, and Yana/UP, Yana/Northern Point). Yana RHS bone collections are being studied by Pavel Nikolskiy.

Yana site - Northern Point Locality (CGG22-CGG28)

Northern Point Locality is the main excavation area studied in 2003 through 2016. Excavations at this site have yielded a large number of bone fragments and artifacts both lithic and organic including a number of mammoth ivory tools, personal ornaments and decorations (Pitulko et al., 2013; Pitulko and Pavlova, 2016). Although not numerous, Pleistocene canid remains (mostly cranial bones, postcranial bones are less frequent) have been found in situ from the occupation level and mapped with exact provenience. All bones have been collected by Pitulko and Pavlova from in situ during the archaeological excavations and directly dated to be c. 32,000 y cal BP. Mitochondrial genomes from seven canid specimens from this site (CGG22-CGG28), have been newly sequenced and analyzed in this study. Three samples (CGG22, CGG25, and CGG28) come from three complete skulls. Three samples (CGG23, CGG24 CGG27) come from partial mandibles (two left and one right). One sample (CGG26) comes from a facial fragment of a canid skull. All samples come from the same layer of the site, but from different units.

Yana site - YMAM locality (CGG14,CGG15, CGG17)

Yana mass accumulation of mammoth, or YMAM is a structural part of the Yana site complex (Pitulko et al., 2013; Pitulko and Pavlova, 2016). *Canis lupus* remains have been collected from the bone-bearing permafrost deposits of the YMAM (Basilyan et al., 2011) by Pitulko and Pavlova in 2008-2012 and dated to c. 30,000 y cal BP, in agreement with multiple radiocarbon dates obtained from the site (see, e.g., Basilyan et al., 2011; Pitulko et al., 2013). Mitochondrial genomes from three canid specimens from this site (CGG14, CGG15 and CGG17), have been newly sequenced and analyzed in this study.

Yana site - SP locality (GGG16)

SP locality is in close proximity to the river bank exposure of YMAM area. The geological sequence is crowned by thick organic-rich Holocene permafrost deposit. A partial canid mandible from this site was collected by V. Pitulko in 2003 (Basilyan et al., 2011). A mitochondrial genome from this specimen (GGG16), radiocarbon dated to be 800 y cal BP has been newly sequenced and analyzed in this study.

Yana site area, Upstream Point locality (CGG18)

Upstream Point is probably the oldest of the localities composing the Yana site complex (Pitulko et al., 2013). Although, numerous lithic artifacts have been collected on the river bank in different years, in situ cultural deposits have never been located at this site. Numerous bone fragments of different Pleistocene

species (examined by Pavel Nikolskiy) have been found eroded from the permafrost deposits that compose the geological sequence of the river bank, including a partial canid mandible (left side) collected by Elena Pavlova and Vladimir Pitulko in 2013. A mitochondrial genome from this canid specimen (CGG18), dated to 41,700 y cal BP, has been newly sequenced and analyzed in this study.

Mus-Khaya exposure (CGG19)

Mus-Khaya exposure is located on the left bank of the Yana River in **northeastern Arctic Siberia**, 5km downstream from the Yana site complex (Pitulko et al., 2011). This exposure is a fragment of high geomorphological surface whose height reaches roughly 50m above the water level (30m in its downstream portion). The Mus-Khaya locality has been known since the late 19th century and actively investigated in the 1960s and 1970s (Katasonov, 2009). It is formed of the Ice Complex deposits of polygenetic origin and as a result, units of different age are recognized in the upper part of its stratigraphic sequence (22,000-18,000; 18,000-14,000; 14,000-10,000 years and Holocene cover). A fragment of Pleistocene wolf mandible (right by body side) was collected by Pitulko and Pavlova in 2012 in the middle part of the exposure on the river bank. A mitochondrial genome from this canid specimen (CGG19), dated to 19,700 y cal BP, has been newly sequenced and analyzed in this study. There is no archaeological context associated with this find.

Bunge-Toll-1885 site (CGG29)

The Bunge-Toll 1885 site locates on the Yana River at N 68° 55’, E 134° 28’ in **northeastern Arctic Siberia** (Pitulko et al., 2014b). The site is named after the Russian Arctic explorers Alexander von Bunge and Eduard von Toll who conducted the expedition dispatched by the Russian Academy of Science and the Russian Geographic Society in 1884-86. The Pleistocene faunal assemblage of this site includes numerous remains of wooly rhinoceros and bison, less frequently reindeer and red deer. Mammoth remains have also been found but are rare. The faunal remains come from the upper part of the permafrost sediments, which fills the bedrock depressions around 50m above the river level. Radiocarbon dating indicates that water-washing by local residents looking for mammoth ivory likely exposed the upper portions of the Yana River’s third terrace sediments. Radiocarbon dating of mammoth, rhinoceros, and bison remains suggests an age of at least 40,000 years (uncalibrated). **There is no known archaeological context related to these finds, however, Pitulko et al. (2016b) report a hunting lesion on a canid humerus suggesting human contact.** A mitochondrial genome from the canid humerus (CGG29), at least 45,000 years old (based on direct radiocarbon dating) (Pitulko et al., 2014b; Pitulko et al., 2016b), has been newly sequenced and analyzed in this study. This sample was collected by Aleksei Tikhonov of Zoological Institute (Russian Academy of Sciences) in 2012.

Nikita Lake site, Muksunuokha River (GGG20)

Nikita Lake site (NKL) is a recently found mass accumulation of mammoth bones that contains the evidence for human involvement in its formation. It is located in the northern part of the Yana-Indighirka coastal lowland on the right bank of the Maksunuokha River under 71°34´56.5˝ N and 141°37´03.5˝ E in 400 m northwest of the northern shore of Nikita Lake in northeastern Arctic Siberia (Pitulko et al., 2016a). It is known since late 1990s. In 2013, it was investigated by Pitulko and Pavlova. It was found that the location is significantly damaged by the ivory mining activities: It has been almost entirely destroyed by the washing out of sediments, performed by local residents mainly in 2011 – 2013. Near the washouts in both, the northern and the southern part, bones of mammoths and other animals are common on the surface. Based on the radiocarbon dating of the faunal remains associated with human activity, people inhabited the site c. 12,000 to 11,800 years ago (uncalibrated). Paleogeographic event sequence reconstruction, based on the available geological data, indicates that during that period humans lived on the shores of the paleo-lake. The evidence of human habitation of this site (faunal remains of mammoth, wolf, wolverine, reindeer, and others, as well as the few stone artifacts) was subsequently picked up by the river and re-deposited into the alluvial sediments and some of these objects were incorporated into erosion channels due to active thermoerosion (Pitulko et al., 2016a). Pleistocene wolf humerus identified by morphology produced one of the youngest radiocarbon ages for this locality – 13,700 y cal BP (Pitulko et al., 2016a). A mitochondrial genome from this specimen (GGG20) has been newly sequenced and analyzed in this study.

Berelekh geoarchaeological complex, Berelekh River (CGG21)

Berelekh is a well-known geoarchaeological complex with a long history of investigation (Pitulko et al, 2011) that starts in 1947 when it was first scientifically described by Grigoriev (Grigoriev, 1957). The site is located on the Berelekh River, a lover left tributary of Indighirka River in northeastern Arctic Siberia, in the middle part of the river valley (70˚ 30' N and 144˚ 02' E), in so-called Ugamyt Tract locality, where two terrace levels can be found. The lower terrace (7m high) formed in the Holocene while the formation of the higher one (12 to 14 m high) is believed to have happened within the Late Pleistocene. The Berelekh geoarchaeological complex belongs to this geomorphic level and includes both mass accumulation of mammoth remains (the 'graveyard') and the 'archaeological site'. Radiocarbon dating of mammoth remains at Berelekh suggests that they were accumulated rapidly during the Bølling warming. Human involvement is unlikely since there is no overlap between radiocarbon dates associated with past human activity, and that of mammoth bone bed. However, lithic artifacts, ivory and bone fragments have been found in sediments exposed on the river bank, next to the bone bed suggesting that humans may have used the mammoth remains after the bone bed was deposited (Pitulko et al., 2014a). Postcranial skeletal elements from canid species have also been found from this exposure. A mitochondrial genome from a canid femur (CGG21) has been newly sequenced and analyzed in this study. The sample has been dated to 14,000 y cal BP (Pitulko et al., 2014a)

Gorky settlement, Ob River (CGG30)

Gorki settlement is located in low Ob River, Western Siberia, Russia (65° 03' N, 65° 17' E), on the right river bank. Concentration of Pleistocene faunal remains was found during the low water stand in the late fall of 2014 by local residents. The bone-bearing level is overlaid by thick sand deposits that make the river terrace, around 15m high. No geological description is available for this place. The assemblage is composed of remains from mammoth, bison, woolly rhinoceros, and a single wolf humerus). The remains were collected by local the local people and handed over to the museum in Salekhard. A mitochondrial genome from the canid humerus (CGG30), radiocarbon dated to be older than 45,000 y cal BP (Pitulko, 2016) has been newly sequenced in this study.

Bibliography

Absolon, K., Klíma, B., 1977. Předmostí Ein Mammutjägerplatz in Mähren. Fontes Archeologiae Moraviae 8.

Basilyan, A.E., M.A. Anisimov, P.A. Nikolskiy, and V.V. Pitulko. 2011. Wooly mammoth mass accumulation next to the Paleolithic Yana RHS site, Arctic Siberia: its geology, age, and relation to past human activity. Journal of Archaeological Science 38, 2461-2474. DOI: 10.1016/j.jas.2011.05.017.

Bocherens, H., Drucker, D.G., Germonpré, M., Lázničková-Galetová, M., Naito, Y.I., Wissing, C., Brůžek, J., Oliva, M., 2015. Reconstruction of the Gravettian food-web at Předmostí I using multi-isotopic tracking (^{13}C, ^{15}N, ^{34}S) of bone collagen. Quaternary International 359-360, 211-228.

Bocherens, H., Drucker, D.G., Bonjean, D., Bridault, A., Conard, N.J., Cupillard, C., Germonpré, M., Höneisen, M., Münzel, S.C., Napierala, H., Patou-Mathis, M., Stephan, E., Uerpmann, H.-P., Ziegler, R. 2011. Isotopic evidence for dietary ecology of cave lion (*Panthera spelaea)* in North-western Europe: prey choice, competition and implications for extinction. Quaternary International 245: 249-261

Bocherens, H., 2015. Isotopic tracking of large carnivore palaeoecology in the mammoth steppe. Quaternary Science Reviews 117: 42-71.

Brace, S., Palkopoulou, E., Dalén, L., Lister, A.M., Miller, R., Otte, M., Germonpré, M., Blockley, S.P.E., Stewart, J.R., Barnes, I., 2012. Serial population extinctions in a small mammal indicate Late Pleistocene ecosystem instability. Proceedings of the National Academy of Sciences 109, 20532-20536.

Charles, R., 1998. Late Magdalenian Chronology and faunal exploitation in the North-Western Ardennes. BAR International Series 737.

De Heinzelin, J., 1980. Archéologie, In A. Gautier and J. de Heinzelin (Eds), La caverne Marie-Jeanne (Hastière-Lavaux, Belgique), Mémoire Institut royal des Sciences Naturelles de Belgique 177, 25-42.

Dupont, E., 1866. Etude sur les fouilles scientifiques exécutées pendant l'hiver de 1865-1866 dans les cavernes des bords de la Lesse. Bulletin de l'Académie royale des Sciences, des Lettres et des Beaux-Arts de Belgique, 2me Série, 22, 31-68.

Dupont, E., 1867. Etude sur cinq cavernes explorées dans la vallée de la Lesse et le ravin de Falmignoul pendant l'été de 1866. Bulletin de l'Académie royale des Sciences, des Lettres et des Beaux-Arts de Belgique, 2me Série, 23, 244-265.

Dupont, E., 1872. L'Homme pendant les âges de la pierre dans les environs de Dinant-sur-Meuse. Bruxelles, Mucquardt.

Ehrenberg, K.,1966. Die Plistozänen Bären Belgiens. III. Teil: Cavernes de Montaigle (Schluss), Cavernes de Walzin, Caverne de Freyr, Cavernes de Pont-à-Lesse. Mémoires Institut royal des Sciences Naturelles de Belgique 155, 1-74.

Gautier, A., 1980. Notes sur les mammifères. In A. Gautier and J. de Heinzelin (Eds), La caverne Marie-Jeanne (Hastière-Lavaux, Belgique), Mémoires Institut royal des Sciences Naturelles de Belgique 177, 25-42.

Germonpré, M., Lázničková-Galetová, M., Sablin, M. 2012. Palaeolithic dog skulls at the Gravettian Předmostí site, the Czech Republic. J. Archaeol. Sc. 39, 184-202.

Germonpré, M., Lázničková-Galetová, M., Losey, R.J., Räikkönen, J., Sablin, M.V., 2015. Large canids at the Gravettian Předmostí site, the Czech Republic: the mandible. Quaternary International 359-360, 261-279

Germonpré, M., Fedorov, S., Danilov, P., Galeta, P., Jimenez, E.-L., Sablin, M., Losey, R.J., 2017. Palaeolithic and prehistoric dogs and Pleistocene wolves from Yakutia: Identification of isolated skulls. J. Archaeol. Sc 78, 1-19.

Giterman, R.E., A.V. Sher, and J.V. Matthews. 1982. Comparison of the development of tundra-steppe environments in west and east Beringia: Pollen and macrofossil evidence from key sections. In: Hopkins D.M, J.V. Matthews, C.E. Schweger, and S.B. Young (eds.). Paleoecology of Beringia. Academic Press: San Diego, CA. pp. 43-73.

Grigoriev, N.F. 1957. Finds of mammoths: Overview of the materials received by the editorial board. Priroda 5, 104–106 (in Russian).

Heierli J. 1907 (ed.). Das Kesslerloch bei Thaingen. Neue Denkschriften der Schweizerischen Naturforschenden Gesellschaft XLIII. Zürcher & Furrer: Zürich.

Höneisen, M., 1986. Kesslerloch und Schweizersbild: Zwei Rentierjäger-Stationen in der Nordschweiz. Archäologie der Schweiz, 9: 28-33.

Jimenez, E.-L., 2016. Palaeoecology and Subsistence Strategies in Belgium and Northwestern Europe during the MIS 3 through the Reassessment of Forgotten Collections: A Methodological Approach. Papers from the Institute of Archaeology, 25, 1-8, DOI: http://dx.doi.org/10.5334/pia-486.

Jimenez, E.-L., Smolderen A., Jadin I., Germonprpre, M., 2016 Exhumation de la collection faunique d'Édouard Dupont provenant du Trou Magrite (Pont-à-Lesse). Quelles données et quelles perspectives pour une collection du XIXe siècle ? *Notae Praehistoricae*, 36/2016 : 167-190

Kandel, A.W., Gasparyan, B., Nahepetyan, S., Taller, A., Weissbrod, L., 2014. The Upper Paleolithic Settlement of the Armenian Highlands. In: M. Otte & F. Le Brun-Ricalens (Eds.) Modes de contacts et de déplacements au Paléolithique eurasiatique, Actes du colloque international de la commission 8 (Paléolithique supérieur) de l'UISPP, Université de Liège, 28-31 mai 2012. ERAUL 140:39-60.

Kaplina, T.N., R.E. Giterman, O.V. Lakhtina, B.A. Abrashov, S.V. Kiselyov, A.V. Sher. 1978. Duvannyy Yar - A key section of Upper Pleistocene deposits of the Kolyma lowland, Bulletin of Quaternary Committe 48, 49–65 (in Russian).

Katasonov, E.M. 2009. Lithology of Frozen Quaternary Deposits (Cryolithology) of the Yana Coastal Lowland. Moscow: PNIIS.

Lee, E.J., D.A. Merriwether, A.K. Kasparov, P.A. Nikolskiy, M.V. Sotnikova, E.Y. Pavlova, and V.V. Pitulko. 2015. Ancient DNA Analysis of the Oldest Canid Species from the Siberian Arctic and Genetic Contribution to the Domestic Dog. PLoS ONE 10(5), e0125759. DOI:10.1371/journal.pone.0125759

Merk, C., 1876. Excavations at the Kesslerloch near Thaygen, Switzerland Longmans, Green and Co. London 65p.

Merk K. 1875. Der Höhlenfund im Kesslerloch bei Thayngen / Originalbericht des Entdeckers. Mittheilungen der Antiquarischen Gesellschaft in Zürich 19: 1–44.

Napierala H. 2008. Die Tierknochen aus dem Kesslerloch - Neubearbeitung der paläolithischen Fauna. Beiträge zur Schaffhauser Archäologie 2. Kantonsarchäologie Schaffhausen: Beiträge zur Schaffhauser Archäologie, pp. 1-128.

Napierala H. & H.-P. Uerpmann, 2010. A „New" Palaeolithic Dog from Central Europe. Int. J. Osteoarchaeol. 2010. doi 10.1002/oa.1182.

Nüesch J. 1904. Das Kesslerloch, eine Höhle aus paläolithischer Zeit / Neue Grabungen und Funde, Neue Denkschriften der allgemeinen Schweizerischen Gesellschaft für die gesammten Naturwissenschaften XXXIX. Zürcher & Furrer, Zürich; 1-72.

Oliva, M., 1997. Les sites pavloviens près de Předmostí. A propos de la chasse au mammouth au Paléolithique supérieur. Acta Mus. Moraviae, Sci. Soc. 82, 3-64.

Pitulko, V.V., P.A. Nikolsky, E.Y. Girya, A.E. Basilyan, V.E. Tumskoy, S.A. Koulakov, S.N. Astakhov, E.Y. Pavlova, M.A. Anisimov. 2004. The Yana RHS Site: Humans in the Arctic before the Last Glaciation. Science 303, 52-56.

Pitulko, V.V. 2011. The Berelekh Quest: A Review of Forty Years of Research in the Mammoth Graveyard in Northeast Siberia. Geoarchaeology 26, 5-32. DOI:10.1002/gea.20342

Pitulko V.V., E.Y. Pavlova, A.E. Basilyan, M. A. Anisimov, P.A. Nikolsky. 2011. Geoarchaeological objects of the low river terraces in the area of the Yana Paleolithic site (Low Yana River, Arctic Siberia), their age and relation to geomorphology and matrix sediments. In: Shurygin, B.N., N.K. Lebedeva, A.A. Goryacheva (eds). Mesosoic and Cenozoic Paleontology, Stratigraphy, and Paleogeography of Boreal Regions. Vol. 2. Novosibirsk: Trofimuk Institute for Oil & Gas Geology. Pp. 133-136.

Pitulko, V., P. Nikolskiy, A. Basilyan, E. Pavlova. 2013. Chapter 2. Human habitation in the Arctic Western Beringia prior the LGM. In K.E. Graf, C.V. Ketron, M.R. Waters (eds). Paleoamerican Odyssey. CSFA, Dept. of Anthropology, Texas A&M University. Pp.13 – 44.

Pitulko, V.V., A. E. Basilyan, E. Y. Pavlova. 2014a. The Berelekh Mammoth Graveyard: New Chronological and Stratigraphical Data from the 2009 field season. Geoarchaeology 29, 277–299.

Pitulko, V.V., A.N. Tikhonov, K.E. Kuper, R.N. Polozov. 2014b. Human-inflicted lesion on a 45,000-year-old Pleistocene wolf humerus from the Yana River, Arctic Siberia. VIth International Conference on Mammoths and their Relatives, Thessaloniki, Greece, May 5th – May 12th, 2014. Abstracts. Scientific Annals of the School of Geology, Aristotle University of Thessaloniki, Greece 102, 156-157.

Pitulko, V.V., and Pavlova, E.Y. 2016. Geoarchaeology and Radiocarbon Chronology of Stone Age Northeast Asia. Center for the Study of the First Americans: Texas A&M University Press, College Station.

Pitulko, V.V. 2016. Evidence for the early human habitation across Arctic Eurasia: new findings and research perspectives. In: Tupakhin, D.S. and N.V. Fedorova (eds). Arctic Archaeology. Vol. 3. Kaliningrad: ID ROS-DOAFK. P. 91-116.

Pitulko, V.V., E.Y. Pavlova, and A.E. Basilyan. 2016a. Mass accumulations of mammoth (mammoth 'graveyards') with indications of past human activity in the northern Yana-Indighirka lowland, Arctic Siberia. Quaternary International 406, 202-217. doi 10.1016/j.quaint.2015.12.039

Pitulko, V.V., A.N. Tikhonov, E.Y. Pavlova, P.A. Nikolskiy, K.E. Kuper, and R.N. Polozov. 2016b. Early human presence in the Arctic: evidence from 45,000-year-old mammoth remains. Science 351, 260-263.

Rougier, H., Crevecoeur, I., Beauval, C., Posth, C., Flas, D., Wißing, C., Furtwängler, A., Germonpré, M., Gómez-Olivencia, A., Semal, P., van der Plicht, J., Bocherens, H., Krause, J., 2016b. Neandertal cannibalism and Neandertal bones used as tools in Northern Europe. Scientific Reports 6, 29005, DOI:10.1038/srep29005

Sher, A.V., T.N. Kaplina, R.E. Giterman, A.V. Lozhkin, A.A. Arkhangelov, S.V. Kiselyov, Y.V. Kouznetsov, E.I. Virina, V.S. Zazhigin. 1979. Late Cenozoic of the Kolyma Lowland. XIV Pacific Science Congress, Tour Guide XI, Khabarovsk August 1979. Moscow, Academy of Sciences of the USSR: XIV Pacific Science Congress, 1-116.

Smolderen, A., 2016. Cinquante nuances de noir. Problèmes de diagnostic en archéologie du feu : études de cas du bassin mosan belge au MIS3. Thèse du grade académique de Docteur en Histoire de l'Art et Archéologie, ULB, Faculté de Philosophie et Sciences sociales.

Svoboda, J. (2008). "The Upper Paleolithic burial area at Předmostí: Ritual and taphonomy." Journal of Human Evolution 54: 15-33.

Vasilchuk, Y.K., A.C. Vasil'chuk, D. Rank, W. Kutschera, J.G. Kim. 2001. Radiocarbon dating of δ 18O- δ D plots in late Pleistocene ice-wedges of the DuvannyYar (lower Kolyma River, northern Yakutia). Radiocarbon 43, 541-553.

Yashina, S., S. Gubin, S. Maksimovich, A. Yashina, E. Gakhova, D. Gilichinsky. 2012. Regeneration of whole fertile plants from 30,000-y-old fruit tissue buried in Siberian permafrost. PNAS 109, 4008-4013.

Zanina, O.G., S.V. Gubin, S.A. Kuzmina, S.V. Maximovich, D.A. Lopatina. 2011. Late-Pleistocene (MIS 3–2) palaeoenvironments as recorded by sediments, palaeosols, and ground-squirrel nests at Duvanny Yar, Kolyma lowland, northeast Siberia. Quaternary Science Reviews 30, 2107–2123.

MOLECULAR ECOLOGY

S2 – DNA Extraction, Sequencing and Bioinformatics.

Here we outline the laboratory and bioinformatics procedures used to generate all new sequences published in this study. In order to achieve a uniform dataset we reprocessed raw reads from previously published samples (from Thalmann et al., 2013 and Skoglund et al., 2015) using the same bioinformatics pipeline as for the newly generated sequences.

2.1 DNA Extractions

We used six different methods to extract endogenous DNA from ancient and modern canids, described below. The method used for each sample is also listed in the supplementary table 1 (column *Extraction*).

1) Eight samples (TU1-TU8) originating from various European excavation sites and dating to the Pleisto- and Holocene were extracted in four batches in clean room facilities of the Royal Belgian Institute for Natural Sciences (RBINS) in Brussels, Belgium. The laboratories are physically separated from any lab in which contemporary material is treated and also from any storage facility of the institute. The ancient labs of the RBINS are pressurized reducing air-influx and equipped with UV-radiation to minimize exogenous DNA contamination. The samples were extracted following the procedure described in Rohland and Hofreiter (2007) and each extraction batch was complemented with a mock sample that contained water instead of actual sample material. In brief, bone or teeth samples were ground with mortar and pestle yielding approx. 50 mg powdered material that was subjected to an overnight lysis. Silica based binding and subsequent washing steps resulted in a final 50 µl eluate that was stored at -20°C until further use.

2) DNA extractions for a total of nine samples (TU9-TU17) were conducted in clean room facilities dedicated to ancient DNA work at the University of Tübingen from 50 mg bone powder per sample. A silica purification protocol was applied as described in Dabney et al. (2013) with the following modifications: the Zymo-Spin V columns (Zymo Research) were UV irradiated for 60 minutes and the total elution volume was raised to 100 µl.

3) A piece of tanned hide from a museum specimen (Ms1) of the Natural History Museum of Denmark was pre-digested for minimally three hours and subsequently fully digested in 1ml buffer as described in Gilbert et al 2007. The supernatant was mixed with 1ml phenol, rotated for 5 min and centrifuged at 3.000 G for 5 min, the aqueous product was mixed with 1 ml chloroform, rotated for 5 min and centrifuged at 3.000 G for 5 min yielding a final aqueous product. The final supernatant was mixed 1:10 with a binding buffer optimized for ancient DNA as detailed in Allentoft et al. (2015) and spun through a

MinElute purification column (Qiagen) attached to an Zymo-Spin extension reservoir (Zymo Research) in a 50 ml falcon tube (Fisher Scientific) as detailed in Dabney et al. 2013, and centrifuge for 30 min at 200 G. The purification column was flowingly washed in 700 µl PE buffer and spun at 8.000 G for 1 min and dry spun at 12.000 G for 2 min. DNA bound to the columns was eluted in two times 25 µl EB buffer after incubation at 37 C for 10 min. All extraction and purification tubes were DNA-lobind (Eppendorf).

4) For 19 samples (CGG13, CGG19-CGG34, Ms6, Ms25), 50-300 mg of crushed bone or tooth was pre-digested in 1 ml buffer as outlined in Ersmark et al 2015. The following steps were identical to procedure 3).

5) 19 (Ms2, Ms4, Ms5, Ms7–Ms22) samples were extracted using the DNeasy Blood & Tissue Kit (Qiagen) following manufacturer's guidelines.

6) Six samples (CGG12, CGG14-CGG18) were digested, extracted and purified using a silica extraction method as described in Allentoft et al. (2015).

2.2 Library preparation

We used three different methods to generate the sequencing libraries. The three methods, described below, vary in the details but are highly comparable as they all build upon an individual barcoding approach. The method used for each sample is also listed in the supplementary table 1 (column *Library_build*).

1) A total of 17 (TU1-TU17) samples were treated the following way. Aliquots of 20 µl extract were converted into double-stranded Illumina libraries using a well-established protocol (Meyer and Kircher, 2010; Kircher et al., 2012). The libraries were quantified before and after the addition of the individual barcodes to monitor the efficiency of the indexing reaction with a quantification assay using the primerset IS7/IS8 and IS5/IS5 (Meyer and Kircher, 2010), the DyNAmo Flash SYBR Green qPCR Kit (Biozym) and the Lightcycler 96 (Roche). The libraries were then amplified using 100 µl reactions for each library containing 5µl library template, 4 units AccuPrime Taq DNA Polymerase High Fidelity (Invitrogen), 1 unit 10X AccuPrime buffer (containing dNTPs) and 0.3 µM IS5 and IS6 primers (Meyer and Kircher, 2010) and the following performed thermal profile: 2-min initial denaturation at 94°C, followed by 4 to 17 cycles consisting of 30-sec denaturation at 94°C, a 30-sec annealing at 60°C and a 2-min elongation at 68°C and a 5-min final elongation at 68°C. After amplification the libraries were purified with the

MinElute PCR purification kit (Qiagen, Hilden, Germany) and quantified using Agilent 2100 Bioanalyzer DNA 1000 chips.

2) For each of 39 samples (CGG13, CGG19-CGG34, Ms1, Ms2, Ms4-Ms22, Ms25), 42,5 µl extract was incorporated into a DNA library using NEBNext DNA Sample Prep Master Mix Set 2 (E6070S - New England Biolabs Inc., Beverly, MA, USA) and Illumina-specific adapters (Meyer and Kircher, 2010). Library build followed producer's guidelines with the following modifications, no initial nebulization step, binding buffer detailed in Allentoft et al. (2015) in the End-repair step, End-repair, adapter ligation and fill-in was performed as in Schroeder et al. (2015) but with 42,5 µl extract as input. Libraries of ancient and historical material were amplified and indexed in reactions of 5 µl library, 32 µl H2O, 1X PCR buffer, 2 µl BSA (20 mg/mL), 1 µl dNTPs (25 mM), 1,5 µl of each of Illumina's Multiplexing PCR primer (1,5 µM of inPE1.0 5'-AATGATACGGCGACCACCGAGATCTACACTCTTTCCCTACACGACGCTCTTCCGATCT and a custom-designed index primer with a six nucleotide index 5'-CAAGCAGAAGACGGCATACGAGATNNNNNNGTGACTGGAGTTC), and 2 µl PfuTurbo Cx Hotstart DNA Polymerase (Agilent Technologies).

Libraries on modern material was amplified and indexed in reactions of 5 µl library, 29,5 µl H2O, 1X PCR buffer, 1 µl dNTPs (25 mM), 2 µM of each of Illumina's Multiplexing PCR primer (same as detailed above), and 0,5 µl Q5 High-Fidelity DNA Polymerase (New England Biolabs Inc). Libraries were purified subsequently over a MinElute (Qiagen) column, following manufacturer's guidelines, eluted in 35 µl EB buffer. DNA concentration in purified amplified libraries was measured using an Agilent 2100 bioanalyzer and pooled in equimolar amounts

3) For six samples (CGG12, CGG14-CGG18) DNA Libraries were built using the NEBNext DNA Sample Prep Master Mix Set 2, following the protocol with modifications. The DNA extract was mixed with 10 µl End Repair buffer and 5 µl End Repair Enzyme Mix and water up to a volume of 100 µl. The mix was incubated for 20 minutes at 12°C and 15 minutes at 37°C, and purified using a Qiagen MinElute PCR Purification Kit. The protocol was followed as directed except that only 30 µl of EB Buffer was used and the column was incubated for 15 minutes at 37°C prior to elution. The eluted DNA was combined with 10 µl NEBNext 5x Quick Ligation Buffer, 5 µl T4 Ligase, and 5 µl P5/P7 Adaptors, diluted to a concentration of 20 uM. This mix was incubated for 20 minutes at 20°C, then purified using a MinElute PCR Purification Kit as described above. 42 µl DNA was eluted and incubated with 5 µl NEBNext Adaptor Fill-In Buffer and 3 µl *Bst* DNA Polymerase for

20 minutes at 65°C and 20 minutes at 80°C. The finished libraries were amplified using Taq Gold in a mix that included 10 µl Taq Gold Buffer, 10 µl $MgCl_2$, 4 µl BSA, 49.2 µl H_2O, 0.8 µl dNTPs, 2 µl of each of Illumina's Multiplexing PCR primer (1.5 µM of inPE1.0 5'-AATGATACGGCGACCACCGAGATCTACACTCTTTCCCTACACGACGCTCTTCCGATCT and a custom-designed index primer with a six nucleotide index 5'-CAAGCAGAAGACGGCATACGAGATNNNNNNGTGACTGGAGTTC), and 2 µl Taq Gold. The PCR conditions were according to manufacturer's directions (10-14 cycles). The PCR reaction was purified using the QIAQuick PCR Purification Kit, with elution of 30 µl EB Buffer and an incubation for 10 minutes prior to the elution step. DNA concentration was assayed using a Qubit 3.0 Fluorimeter, following manufacturer's instructions. If the DNA concentration was less than 20 ng/µl, a second PCR amplification was performed using Phusion. The mix included 20 µl template DNA, 2 µl each of primers IS5 and IS6, 50 µl Phusion Master Mix, and 26 µl H_2O. The PCR program followed manufacturer's instructions but for 6-10 cycles, and was purified with a QIAQuick PCR Purification Kit as described above. The DNA concentration of the libraries was assessed using an Agilent 2100 Bioanalyzer.

2.3 Sequence generation

We used three different methods to generate full mitochondrial genome sequences. The method used for each sample is also listed in the supplementary table 1 (column *Sequence generation*).

1) Seventeen samples (TU1-TU17) were enriched for the dog mitochondrial genome using the amplified libraries, pooled in equimolar amounts, and bead capture enrichment as detailed elsewhere (Maricic et al., 2010; Thalmann et al., 2013). After enrichment the libraries were amplified in 100 µl reactions with 15 µl template, 2 units Phusion High Fidelity DNA polymerase, 1 unit 5x HF buffer, 0.25 mM dNTPs and 0.3 µM IS5 and IS6 primers, and the following thermal profile: 5-min initial denaturation at 95°C, followed by 16 to 23 cycles consisting of 30-sec denaturation at 95°C, a 30-sec annealing at 60°C and a 45-sec elongation at 72°C and a 5-min final elongation at 72°C. Finally, the libraries were purified using MinElute columns (Qiagen, Hilden, Germany), quantified with Agilent 2100 Bioanalyzer DNA 1000 chips and diluted to 10 nM for sequencing. Sequencing was conducted with a paired-end dual index run on an Illumina Hiseq 2500 platform with 2*100+7+7 cycles and the manufacturer's protocols for multiplex sequencing (TruSeq PE Cluster Kit v3-cBot-HS).

2) 'Shot-gun' sequences were generated for a total of 39 samples (CGG13, CGG19-CGG34, Ms1, Ms2, Ms4-Ms22, Ms25) on an Illumina HiSeq 2500 platform, using 100 bp single

read chemistry for ancient and historical material and 200 bp paired end read chemistry for modern samples.

3) Six libraries (CGG12, CGG14-CGG18) were captured using a Mycroarray MYBaits kit, which included custom RNA probes that corresponded to 4x tiling of the dog mitogenome. The capture was performed according to manual version 2.3.1, with a hybridization time of 18 hours and a temperature of 65°C. Following capture, the DNA was amplified using KAPA HI-Fi HotStart DNA Polymerase, using a mix of 15 µl captured DNA, 25 µl KAPA Hi-Fi HotStart Master Mix, 0.75 µl primers IS5 and IS6 at a concentration of 10 µM, and 8.5 µl H2O. The thermocycler program corresponded to manufacturer's instructions, and the samples were amplified for 14 cycles and then purified with a QIAQuick PCR Purification Kit as previously described. The DNA concentration of the captures was assessed using an Agilent 2100 Bioanalyzer, then they were pooled in equimolar amounts and sequenced on a HiSeq 2500 with 100 bp single read chemistry.

2.4 Raw Sequence Data Processing

Raw sequencing reads of all newly generated samples were de-tagged from their individual barcodes and cleaned of all adapters needed for sequencing. A merging procedure was applied to sequences generated with paired-end technology (sample codes beginning with "TU") as detailed in Thalmann et al. (2013). In brief, all forward and reverse reads of each sequenced individual were merged into a single read based on overlap identity and all other, un-merged reads were excluded. Sequences generated with single-end technology (CGG) were processed individually.

Two assembly protocols were adapted and the resulting consensus sequences compared for conformity.

1) All merged reads and single-end reads were implemented into a reference guided, iterative assembly employing the program MIA (Green et al., 2008). The following command was used:

mia –r NC_002008.fas –f SAMPLE.fq –c –C –U –F –k 12 –s ANCIENT_SUBMAT.txt –m SAMPLE_OUT.maln

As reference (-r) served the dog mt-genome (NC_002008, Kim et al., 1998). SAMPLE.fq (-f) indicates all merged reads for each individual in .fastq format and a scoring matrix (-s) for aligning sequencing reads generated from ancient materials (ANCIENT_SUBMAT.txt) was also applied. Other parameters used here can be found in the MIA documentation. A consensus sequence was called for each individual with default parameters but only positions with a min of 3-fold coverage were retained in the final consensus sequence. This is a difference to the approach in Thalmann et al. (2013) and sequences generated in there were newly filtered for 3x coverage in order to further eliminate ambiguous positions. Moreover, we omitted a comprehensive screen for nuclear-mitochondrial inserts (Ishiguro et al., 2002, Thalmann et al., 2013) since previous

analyses have shown that only a negligible small number of reads generated from similar materials mapping preferentially to the nuclear genome of the dog.

2) A second mapping approach using the methods outlined below did not reveal significant differences in the generated consensus sequences. Paleomix (Schubert et al., 2014) was used to align the reads of 19 samples against the dog reference mitochondria genome (NC_002008). Illumina reads were trimmed using default settings in AdapterRemoval2 (Schubert et al., 2016), except using a minimal read length of 25bp. Cleaned reads were inspected for quality control using FastQC (http://www.bioinformatics.babraham.ac.uk/projects/fastqc) and aligned using BWA (Li & Durbin 2009), disabling the seed (Schubert et al., 2012). Mapped reads to multiple positions and with mapping quality scores inferior to 30 were removed using SAMtools (Li et al., 2009). Sequence duplicates were removed using MarkDuplicates from Picard (http://www.broadinstitute.github.io/picard). The final alignment was realigned and SNPs were called using GATK (McKenna et al., 2010) and filtered using Vcftools (Danecek et al., 2011). Consensus sequences were made in two ways using GATK. One way was using SNPs that passed the majority count threshold and the other adding IUPAC bases where GATK called as "heterozygous" site (McKenna et al., 2010). Mapping and coverage statistics were computed using Paleomix (Schubert et al., 2014) internal scripts. This previous methodology was also applied to the 1 million reads subset in order to compare them with other samples.

2.5. Molecular characteristics of newly generated, ancient sequences

In order to assess the quality and molecular characteristics of the newly generated ancient sequences we performed additional analyses. We remapped all reads generated in capture experiments against the complete dog genome (canFam3) following the method outlined in SI2.4.2. We extracted all quality filtered reads, removed PCR duplicates and investigated the fraction of filtered reads that mapped exclusively against the mitochondrial genome. We only retained read pairs generated with paired-end sequencing technology that were collapsed into a single read (merged). Table S2 provides a summary of this analysis. Samples generated using a shot-gun approach (CGG) generally deliver a higher fraction of reads successfully mapped, however, the target specificity towards the small mitochondrial genome is low. This can be increased by using DNA capture procedures (Gnirke et al. 2009). While in the shot-gun approach the fraction of reads mapping exclusively to the dog mitochondrial genome ranges between 0.005%-0.549% of all filtered reads mapping successfully to the complete dog genome, this ratio is increased to almost 100% (see Table S2) for captured mitochondrial DNA fragments. On average, capture approaches gave a 500-fold increase in the fraction of reads mapping exclusively to the dog mitochondrial genome. Interestingly, the efficiency of different capture methods varies as evidenced by the higher percentage of filtered reads mapping against the dog mitochondrial genome for the re-captured CGG samples (CGG12-CGG18 all above 85%, values in brackets in Table S2, see SI2.3.3) compared to those generated from samples TU (2.4%-98.66%, lower panel in Table S2, see SI2.3.1).

There is no apparent correlation between sample age and fragment size (Fig. S1) or deamination patterns (Fig. S2). Some of the longest fragments were retrieved from samples approximately 30,000 to 46,000 years old (CGG18, TU1, TU2, TU3, TU7) while shorter fragments were observed in younger samples (CGG20, TU14, TU4). Intriguingly, TU1, TU2, TU3, TU7 and the younger TU8 originated from caves in Belgium (Table SI1). Furthermore, previous findings suggested that the frequency of C to T misincorporations at the 5' end (or G to A misincorporations at the 3' end) of ancient molecules increases with age of the sample (Sawyer et al. 2012). However, the ancient DNA fragments newly generated for this study do not show any significant trend with sample age (Fig. S2, $p \geq 0.45$). Taken together this points towards factors other than the sample age playing an important role in shaping the characteristics of ancient molecules.

Table S2

	Sequencing	Mapping		
Sample	# reads total	# filtered reads mapping the complete dog genome	# filtered reads mapping exclusively the dog mt-genome	% filtered reads mapping the dog mt-genome
CGG12	20,502,189 (8,795,253)	1,461,472 (22,120)	581 (21,779)	0.040 (98.46)
CGG13	10,454,317 (9,837,169)	2,921,726 (35,942)	157 (30,711)	0.005 (85.45)
CGG14	29,231,546 (16,073,166)	304,327 (18,194)	1,394 (18,142)	0.458 (99.71)
CGG15	17,563,376 (5,028,725)	526,430 9,776)	419 (9,726)	0.080 (99.49)
CGG16	8,426,724 (6,982,988)	188,881 (24,367)	53 (23,740)	0.028 (97.43)
CGG17	27,612,032 (15,525,823)	3,406,363 (25,846)	1,224 (25,350)	0.036 (98.08)
CGG18	5,342,504 (8,845,755)	530,495 (23,048)	92 (22,421)	0.017 (97.28)
CGG19	44,387,927	2,747,206	2,227	0.081
CGG20	30,039,526	2,806,030	1,852	0.066
CGG21	31,161,726	6,880,522	3,014	0.044
CGG22	14,125,908	467,594	2,568	0.549
CGG23	20,899,573	14,788,539	767	0.005
CGG24	17,223,554	75,513	248	0.328
CGG25	13,204,816	7,208,601	2,883	0.040
CGG26	28,498,152	2,828,408	1,856	0.066
CGG27	28,947,324	22,295,797	4,696	0.021
CGG28	16,918,816	4,376,379	1,945	0.044
CGG29	40,530,428	23,217,595	5,728	0.025
CGG30	89,127,852	246,756	310	0.126
CGG31	10,524,144	895,714	1,948	0.217
CGG32	26,685,260	14,231,440	2,196	0.015
CGG33	24,737,165	18,241,333	5,500	0.030
CGG34	14,776,135	6,668,532	1,153	0.017
TU1	1,071,820	4,943	539	10.90
TU2	940,066	6,676	610	9.14

TU3	1,424,226	19,367	539	2.78
TU4	6,963,257	78,775	1,892	2.40
TU5	1,437,297	46,883	1,251	2.67
TU6	2,383,565	50,063	15,448	30.86
TU7	5,193,206	218,867	9,050	4.13
TU8	3,378,610	329,599	28,182	8.55
TU9	264,033	3,493	3,248	92.99
TU10	439,293	12,748	12,577	98.66
TU11	4,393,084	46,075	30,095	65.32
TU12	856,459	1,135	502	44.23
TU13	4,607,297	4,974	1,697	34.12
TU14	852,538	7,933	3,017	38.03
TU15	1,063,036	1,943	1,213	62.43
TU16	4,957,011	4,594	801	17.44
TU17	2,330,648	1,262	767	60.78

Table S2: Proportion of sequencing reads mapped against the dog nuclear genome and mitochondrial genomes. The upper panel shows data generated with single-end sequencing technology and the lower panel (TU) of those produced with paired-end technology.

Numbers in brackets represent data generated using mt-genome capture technique (described in SI2.3.3). Samples highlighted in red were not included in the demographic analyses (Table SI1).

MOLECULAR ECOLOGY

Figure S1

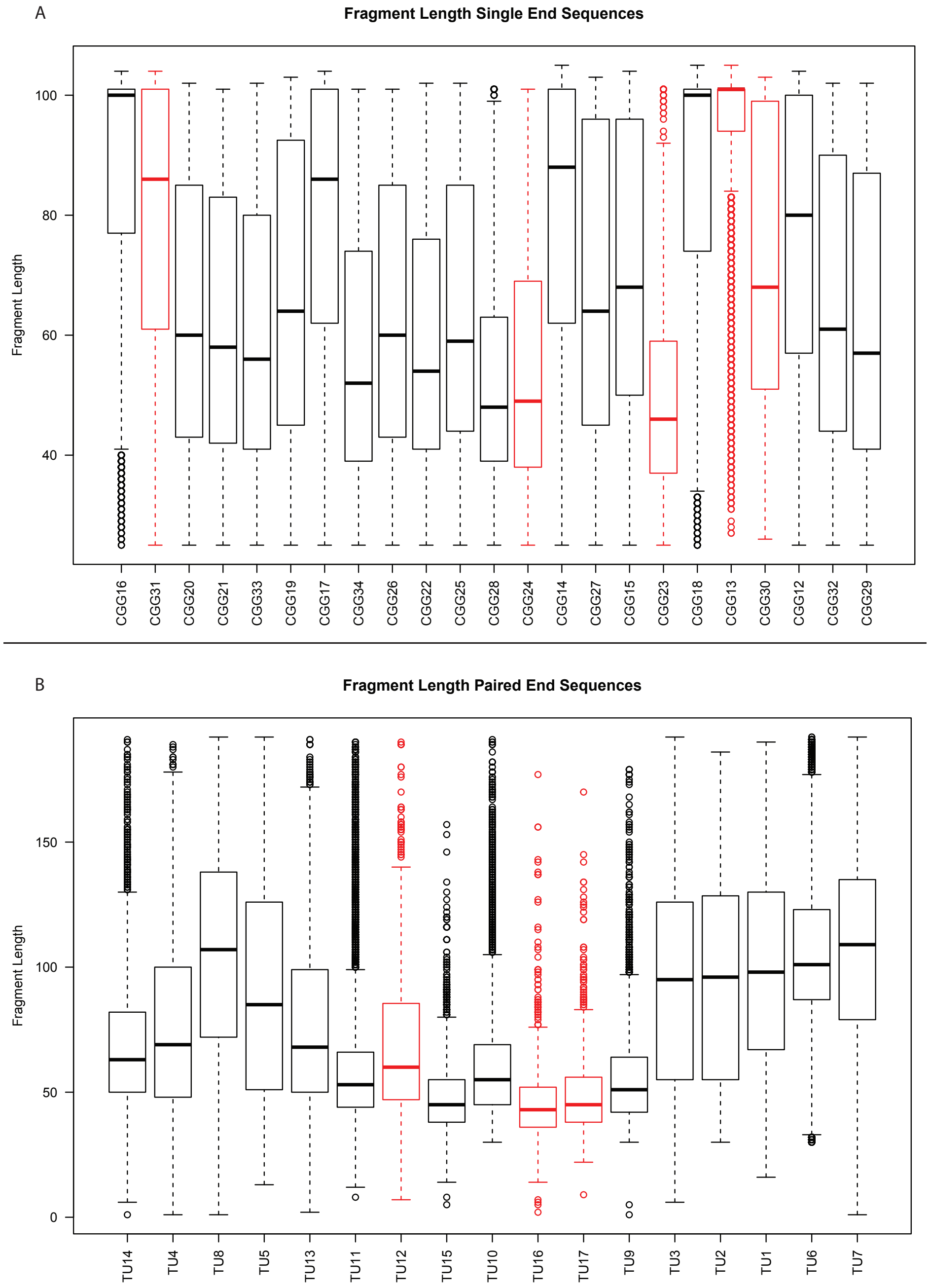

Figure S1. Fragment length distribution for all newly generated, ancient sequences. (A) Length of the fragments generated with single-end sequencing technology. (B) Length of the fragments generated with paired-end sequencing technology. Samples excluded from demographic analyses are highlighted in red. Samples are ordered by age with the youngest on the left.

MOLECULAR ECOLOGY

Figure S2

A

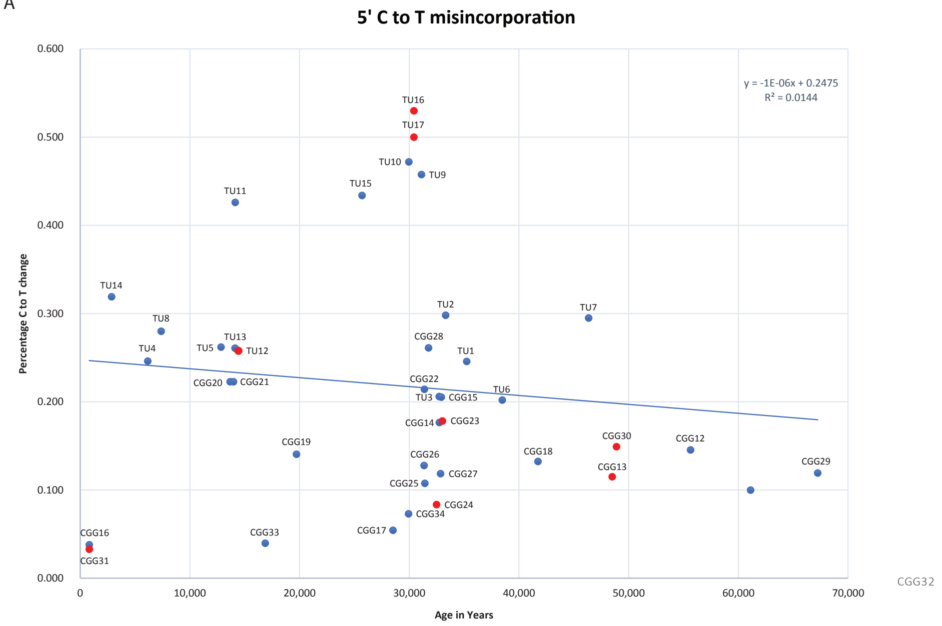

B

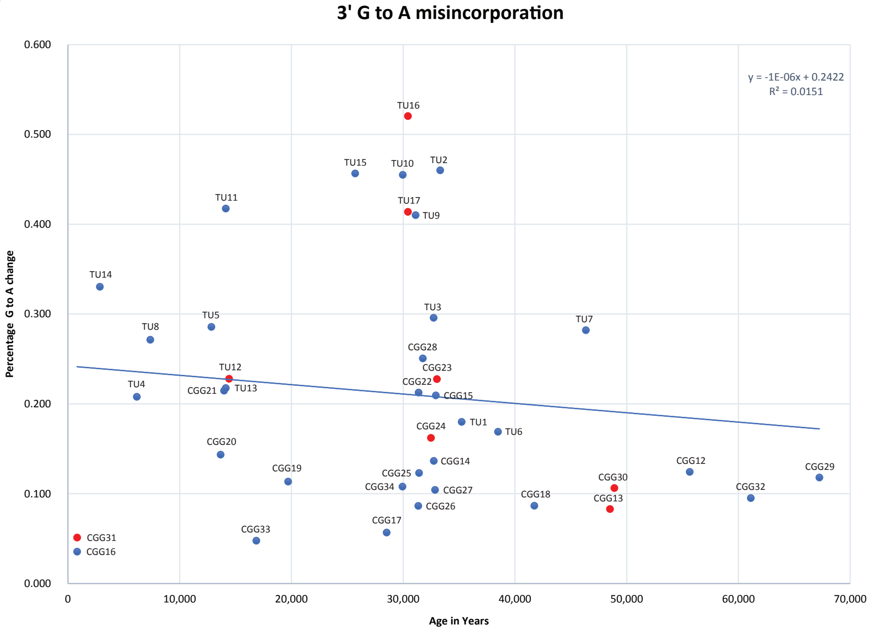

Figure S2. Deamination patterns at the respective start bases of the newly generated, ancient fragments as a function of sample age. (A) C to T misincorporation frequency at the first base of the 5' end of the fragments. (B) G to A misincorporation frequency at the first base of the 3' end of the fragments. The blue lines represent the best linear fit. Samples excluded from demographic analyses are highlighted in red.

Bibliography:

Allentoft, M. E. et al. 2015. Population genomics of Bronze Age Eurasia. *Nature* 522, 167-172.

Andrews, S., 2010. FastQC: a quality control tool for high throughput sequence data. (available at http://www.bioinformatics.babraham.ac.uk/projects/fastqc).

Broad Institute, Picard. A set of command line tools (in Java) for manipulating high-throughput sequencing (HTS) data and formats such as SAM/BAM/CRAM and VCF, (available at http://broadinstitute.github.io/picard/).

Dabney, J. et al. 2013. Complete mitochondrial genome sequence of a Middle Pleistocene cave bear reconstructed from ultrashort DNA fragments. *Proceedings of the National Academy of Sciences USA* 110, 15758-15763.

Danecek, P. et al. 2011. The variant call format and VCFtools. *Bioinformatics*. 27, 2156–8.

Ersmark, E. et al. 2015. Population demography and genetic diversity in the Pleistocene cave lion. *Open Quaternary*, 1, DOI: http://doi.org/10.5334/oq.aa.

Gilbert, M. T. P. et al. 2007. Whole-genome shotgun sequencing of mitochondria from ancient hair shafts. *Science* 317, 1927-1930.

Gnirke, A., et al. 2009. Solution hybrid selection with ultra-long oligonucleotides for massively parallel targeted sequencing. *Nature biotechnology* 27.2, 182-189.

Green, R. E. et al. 2008. Complete Neandertal mitochondrial genome sequence determined by high-throughput sequencing. *Cell* 134, 416–426.

Ishiguro, N., Nakajima, A., Horiuchi, M. & Shinagawa, M., 2002. Multiple nuclear pseudogenes of mitochondrial DNA exist in the canine genome. *Mammalian Genome* 13, 365–372.

Kim, K. S., Lee, S. E., Jeong, H. W. & Ha, J. H., 1998. The complete nucleotide sequence of the domestic dog (Canis familiaris) mitochondrial genome. *Molecular Phylogenetics and Evolution* 10, 210-220.

Kircher, M., Sawyer, S. & Meyer, M., 2012. Double indexing overcomes inaccuracies in multiplex sequencing on the Illumina platform. *Nucleic Acids Research* 40, e3

Kircher, M., 2012. in Ancient DNA (eds. Shapiro, B. & Hofreiter, M.) 840, 197–228 (Humana Press)

Li, H. & Durbin, R., 2009. Fast and accurate short read alignment with Burrows-Wheeler transform. *Bioinformatics* 25, 1754–60.

Li, H. et al. 2009. The Sequence Alignment/Map format and SAMtools. *Bioinformatics* 25, 2078–9.

Meyer, M. & Kircher, M., 2010. Illumina sequencing library preparation for highly multiplexed target capture and sequencing. *Cold Spring Harbor Protocols*, pdb.prot5448.

Maricic, T., Whitten, M. & Pääbo, S., 2010. Multiplexed DNA sequence capture of mitochondrial genomes using PCR products. *PLoS ONE* 5, e14004.

McKenna, A. et al. 2010. The Genome Analysis Toolkit: a MapReduce framework for analyzing next-generation DNA sequencing data. *Genome Research* 20, 1297–303.

Sawyer, S. et al. 2012. Temporal patterns of nucleotide misincorporations and DNA fragmentation in ancient DNA. *PLoS ONE* 7, e34131.

Schroeder, H. et al. 2015. Genome-wide ancestry of 17th-century enslaved Africans from the Caribbean. *Proceedings of the National Academy of Sciences USA* 112, 3669-3673.

Schubert, M. et al. 2012. Improving ancient DNA read mapping against modern reference genomes. *BMC Genomics*. 13, 178.

Schubert, M. et al. 2014. Characterization of ancient and modern genomes by SNP detection and phylogenomic and metagenomic analysis using PALEOMIX. *Nature Protocols* 9, 1056–1082.

Schubert, M., Lindgreen, S. & Orlando, L., 2016. AdapterRemoval v2: rapid adapter trimming, identification, and read merging. *BMC Research Notes* 9, 1–7.

Skoglund, P., Ersmark, E., Palkopoulou, E. & Dalén, L., 2015. Ancient Wolf Genome Reveals an Early Divergence of Domestic Dog Ancestors and Admixture into High-Latitude Breeds. *Current Biology*, 1515–1519.

Rohland, N. & Hofreiter, M., 2007. Ancient DNA extraction from bones and teeth. *Nature Protocols* 2, 1756–1762.

Thalmann, O. et al. 2013. Complete Mitochondrial Genomes of Ancient Canids Suggest a European Origin of Domestic Dogs. *Science* 342, 871-874.

MOLECULAR ECOLOGY

S3 – Data Analyses & Results

3.1 BEAST analyses & results

We used the BEAST tool (v.1.8.0) (Drummond et al., 2012) to build a tip calibrated wolf mitochondrial tree and to estimate mutation rates for the four different partitions of the wolf mitochondrial genome.

Partition finder

We used the PartitionFinder tool (Lanfear et al., 2012) and a subset of the gray wolf mitochondrial genome alignment containing 73 modern wolf samples (see supplementary table 1) to fit a substitution model to the partitions of the mitochondrial genome. The 6 partitions were based on the biological properties of the mammalian mitochondrial genome and defined as the 3 codons of the protein coding sequence (PCS1, PCS2 and PCS3), rRNA, tRNA and the D-Loop. The positions of the partitions in the sequence were established using an annotated dog mitochondrial sequence as a reference (Genbank accession nr NC_002008 (Kim et al., 1998)).

The “branch lengths” parameter was set as “linked”

The “model of evolution” parameter was set as “all”

The “model selection” parameter was set as “BIC”

The “search schemes” parameter was set as “greedy”

The best fitting mutation scheme suggested four partitions with independent mutation models listed in the Table S3

Table S3

Partition	Model
PCDS1, rRNA, tRNA	HKY+I
PCDS2	TrN+I
PCDS3	TrN+G
D-loop	TrN+I+G

Table S3: The best fitting mutation scheme from Partition Finder.

Mutation rate calculation

In order to estimate the grey wolf mitochondrial mutation rate, we combined modern grey wolf mitochondrial genome sequences (N = 78) with mitochondrial genome sequences from 38 ancient, directly radio carbon dated samples. All the sequences were subjected to strict quality criteria (see above). The ages (in years before present) of ancient samples in the BEAST analyses were set as a mean of calibrated radiocarbon age (years before present) distribution. See supplementary table 1 for list of samples and their radiocarbon ages included in the mutation mitochondrial rate estimation.

Input file settings

All input files for the BEAST analyses were created using the BEAUti (v.1.8.0) tool (Drummond et al., 2012) with default parameter settings, unless specified otherwise.

Based on the estimated best fitting partitioning scheme by PartitionFinder (see above), the mitochondrial genome was represented in the BEAST input file as four independent partitions: 1) tRNA & rRNA; 2) PCDS1; 3) PCDS2, 4) PCDS3 (defined as above)

To represent the fact that the mitochondrion is a single non-recombining locus, the tree models for all four partitions were linked while the site and the clock models for the four partitions were set as unlinked between the four partitions.

The substitution model parameters for the four partitions were fixed to the ones estimated by Partition finder (see above).

Our samples span 60 thousand years – a short time in an evolutionary scale - and come from a single species. As a result we used a strict (global) clock, which assumes no rate variation among different linages of the tree. However, relaxing the clock rate parameter had no measurable effect on the estimates. To allow for changes in population size through time, the Coalescent Bayesian Skyline (Drummond et al., 2005) was used as the tree prior, with a group size parameter set as 20.

Due to the fact that ancient samples are known to carry excess transition, we performed two independent BEAST runs: 1) where we did not use any sequence error model and 2) where we accounted for the potential the sequence error by using age-dependent (transition only) model. However, there was no noticeable difference between the estimates of the two runs, probably reflecting the strict quality criteria we subjected our sequences to. As a result, we did not include any sequence error model in subsequent runs.

Priors

A lognormal distribution with an offset of 0.0, a (log) mean of 1.0 and a (log) standard deviation of 1.25 was used as the *kappa* prior for all four partitions.

A uniform distribution with a lower bound of 0.0 and upper bound on 1.0 was used as *frequencies* prior for all four partitions.

A lognormal distribution with an offset of 0.0, a (log) mean of -18.42068 (corresponding to 1E-8 in real space) and a (log) standard deviation of 1.5 was used as the *clock rate* prior for all four partitions.

A gamma distribution with an offset of 0.0, a shape of 1.0 and a scale of 100,000 was used as the *root height* parameter. The gamma distribution was truncated using a lower bound of 50,000 and upper bound of 2,000,000.

A gamma distribution with an offset of 0.0, a shape of 2.0 and a scale of 100,000 was used as the *skyline population size* prior.

MCMC chain

For all runs the model parameters and trees were sampled every 5,000 iterations over 50,000,000 iterations. The first 10% of the recorded iterations were discarded as a burn-in period.

BEAST results

The MCMC chain convergence for all parameters was assessed using the Tracer (v1.6) program (Rambaut et al., 2014) and the sampled trees were summarized and the maximum clade credibility tree calculated using the program TreeAnnotator (v 1.8.0) (Rambaut and Drummond 2013).

For mitochondrial clock rate estimates see table S4. For other parameter estimates, ESS values and convergences a BEAST log file is available from L.L. upon request.

Table S4

Partition	Mean (Rate)	ESS	95% HPD interval
tRNA_rRNA	3.38E-08	2119	[2.6621E-8, 4.0527E-8]
PCDS1	4.51E-08	2003	[2.6621E-8, 4.0527E-8]
PCDS2	4.73E-08	1809	[3.8539E-8, 5.6882E-8]
PCDS3	1.12E-07	1245	[9.5106E-8, 1.2969E-7]

Table S4: Mitochondrial clock rate estimates from BEAST

Table S5

Sample ID	Mean (Age in Years BP)	ESS	95% HPD interval
TH4	52153.715	520	[39259.1785, 65899.259]
TH6	29272.436	3572	[21900.1268, 36390.9274]
TH14	4802.368	7714	[-1077.3547, 11121.1716]
TU15	25694.856	6908	[16597.0261, 34901.8937]
CGG12	55643.388	645	[43151.1409, 68838.1615]
CGG29	67239.038	417	[50579.1862, 80836.7417]
CGG32	61110.94	450	[48920.877, 73682.795]

Table S5: BEAST estimated molecular dates.

3.2 Spatial Modelling Results

Table S6

Model	Marginal Density	Nr Retrained	Likelihood	Bayes factor
Static	4.36E-09	535984	2.34E-03	0.125
Bottleneck	5.34E-09	487140	2.60E-03	0.139
Expansion_Europe	4.48E-09	747804	3.35E-03	0.179
Expansion_Central_North_Eurasia	3.45E-09	1821779	6.29E-03	0.337
Expansion_Beringia	3.71E-09	3467674	1.29E-02	0.689
Expansion_Middle-East	4.08E-09	1047509	4.28E-03	0.229
Expansion_East-Eurasia	3.47E-09	2613115	9.07E-03	0.486
Expansion_Arctic_North-America	6.95E-09	1089710	7.58E-03	0.406
Expansion_North-America	7.00E-09	1092975	7.65E-03	0.410
Bottleneck_Expansion_Europe	5.42E-09	786952	4.27E-03	0.229
Bottleneck_Expansion_Central_North_Eurasia	5.06E-09	1733133	8.77E-03	0.470
Bottleneck_Expansion_Beringia	5.92E-09	3155396	1.87E-02	1.00
Bottleneck_Expansion_Middle-East	5.35E-09	1077919	5.77E-03	0.309
Bottleneck_Expansion_East-Eurasia	5.15E-09	2430622	1.25E-02	0.671
Bottleneck_Expansion_Arctic_North-America	6.46E-09	612690	3.96E-03	0.212
Bottleneck_Expansion_North-America	6.35E-09	613120	3.89E-03	0.209

Table S6: ABC likelihoods and Bayes factors for all demographic scenarios tested

Table S7

OUT OF BERINGIA	log_{10} m	log_{10} K_1	log_{10} K_2	log_{10} K_3	X	T	ΔT
mode	-1.26209	0.626259	-0.949534	0.383645	0.212121	24.0908	0.212909
mean	-0.290149	0.110089	-0.148737	-0.0562393	0.498742	0.678518	0.498851
median	-1.25014	0.514361	-0.664665	-0.170467	0.491903	23.8563	0.491049
lower_95_quantile	-1.94567	-1.3756	-1.90336	-1.851	0.0344065	13.0187	0.035245
upper_95_quantile	-0.535434	1.78503	1.12646	1.32106	0.956443	33.8313	0.956443
HPD_95_lower	-1.91358	-1.15146	-1.9999	-1.91909	0.04052	13.485	0.041431
HPD_95_upper	-0.56717	1.91913	0.828077	1.19155	0.949378	33.9896	0.959569

Table S7: Posterior probability estimates for seven estimated parameters (ΔT, T, log_{10} K_1, log_{10} K_2, log_{10} K_3, log_{10} m, X) in the most likely model (Expansion out of Beringia with a population size change)

Table S8

OUT OF EAST EURASIA	log_{10} m	log_{10} K_1	log_{10} K_2	log_{10} K_3	X	T	ΔT
mode	-1.21848	0.626262	-0.626269	0.382251	0.616161	23.3838	0.223
mean	-0.280727	0.117214	-0.11224	-0.0599404	0.503048	0.662603	0.498211
median	-1.21217	0.543323	-0.513207	-0.190711	0.498322	23.3253	0.48959
lower_95_quantile	-1.89641	-1.366	-1.88733	-1.85371	0.0355581	10.7056	0.0351268
upper_95_quantile	-0.487195	1.8011	1.40089	1.31595	0.957555	34.6578	0.956376
HPD_95_lower	-1.86995	-1.03017	-1.9999	-1.91914	0.0404699	11.3638	0.0414806
HPD_95_upper	-0.523578	1.95946	1.111	1.18969	0.95953	34.6967	0.949427

Table S8: Posterior probability estimates for seven estimated parameters (ΔT, T, log_{10} K_1, log_{10} K_2, log_{10} K_3, log_{10} m, X) in the most likely model (Expansion out of East Eurasia with a population size change)

Bibliography:

Drummond, A. J., A. Rambaut, B. Shapiro, and O. G. Pybus. 2005. Bayesian Coalescent Inference of Past Population Dynamics from Molecular Sequences. *Molecular Biology and Evolution* 22 (5): 1185–92. doi:10.1093/molbev/msi103.

Drummond, Alexei J., Marc A. Suchard, Dong Xie, and Andrew Rambaut. 2012. Bayesian Phylogenetics with BEAUti and the BEAST 1.7. *Molecular Biology and Evolution* 29 (8): 1969–73. doi:10.1093/molbev/mss075.

Kim, K. S., S. E. Lee, H. W. Jeong, and J. H. Ha. 1998. The Complete Nucleotide Sequence of the Domestic Dog (Canis Familiaris) Mitochondrial Genome. *Molecular Phylogenetics and Evolution* 10 (2): 210–20. doi:10.1006/mpev.1998.0513.

Lanfear, Robert, Brett Calcott, Simon Y. W. Ho, and Stephane Guindon. 2012. PartitionFinder: Combined Selection of Partitioning Schemes and Substitution Models for Phylogenetic Analyses. *Molecular Biology and Evolution* 29 (6): 1695–1701. doi:10.1093/molbev/mss020.

Rambaut, A., and A. J. Drummond. 2013. *TreeAnnotator*.

Rambaut, A., M. A. Suchard, D. Xie, and A. J. Drummond. 2014. *Tracer* (version 1.6.).

MOLECULAR ECOLOGY

3.3 Supplementary figures

Figure S3

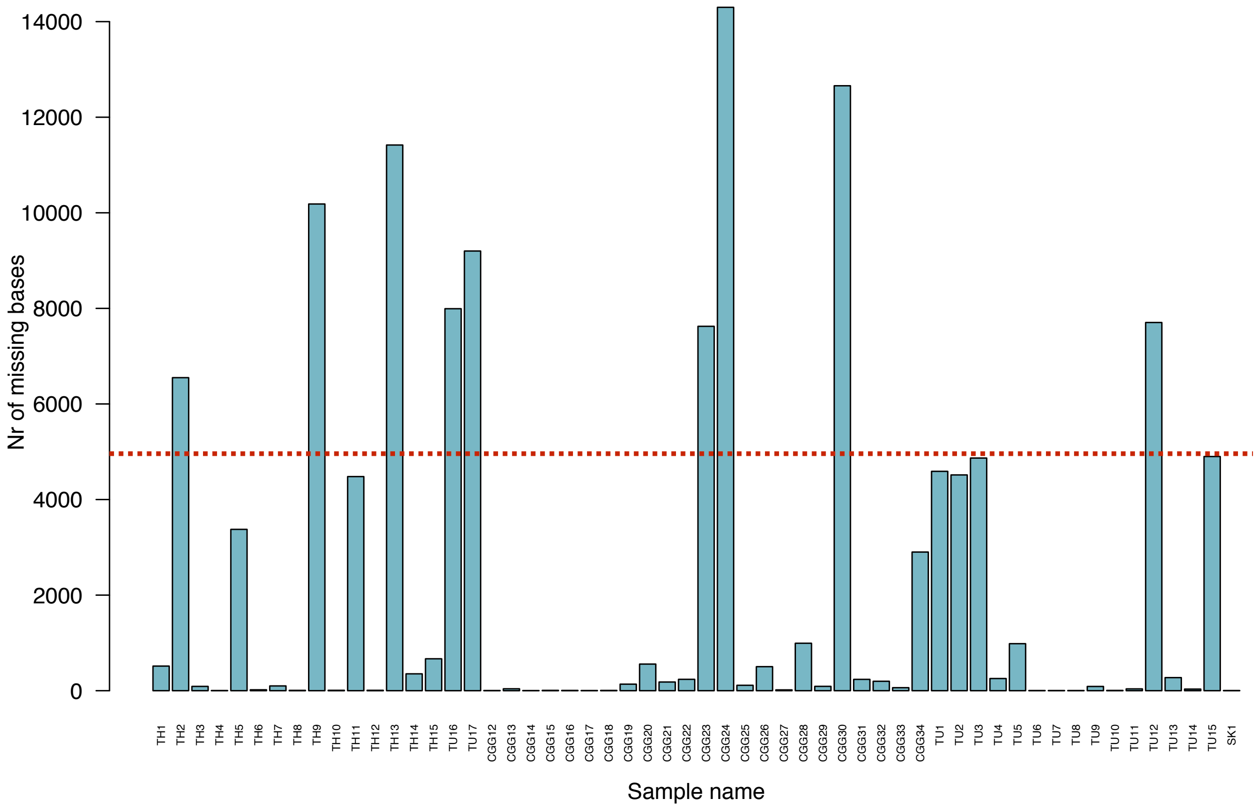

Figure S3: Number of bases missing from the complete mitochondrial genome (total of 15,463 base pairs - excluding the control region) (on y-axis) for each of the samples (x-axis). Nine sequences had more than 1/3 (5,150 bases) missing and were therefore excluded from all analyses (See supplementary table 1 for list of excluded samples). The red line represents the cut-off point (5,150 bases).

MOLECULAR ECOLOGY

Figure S4

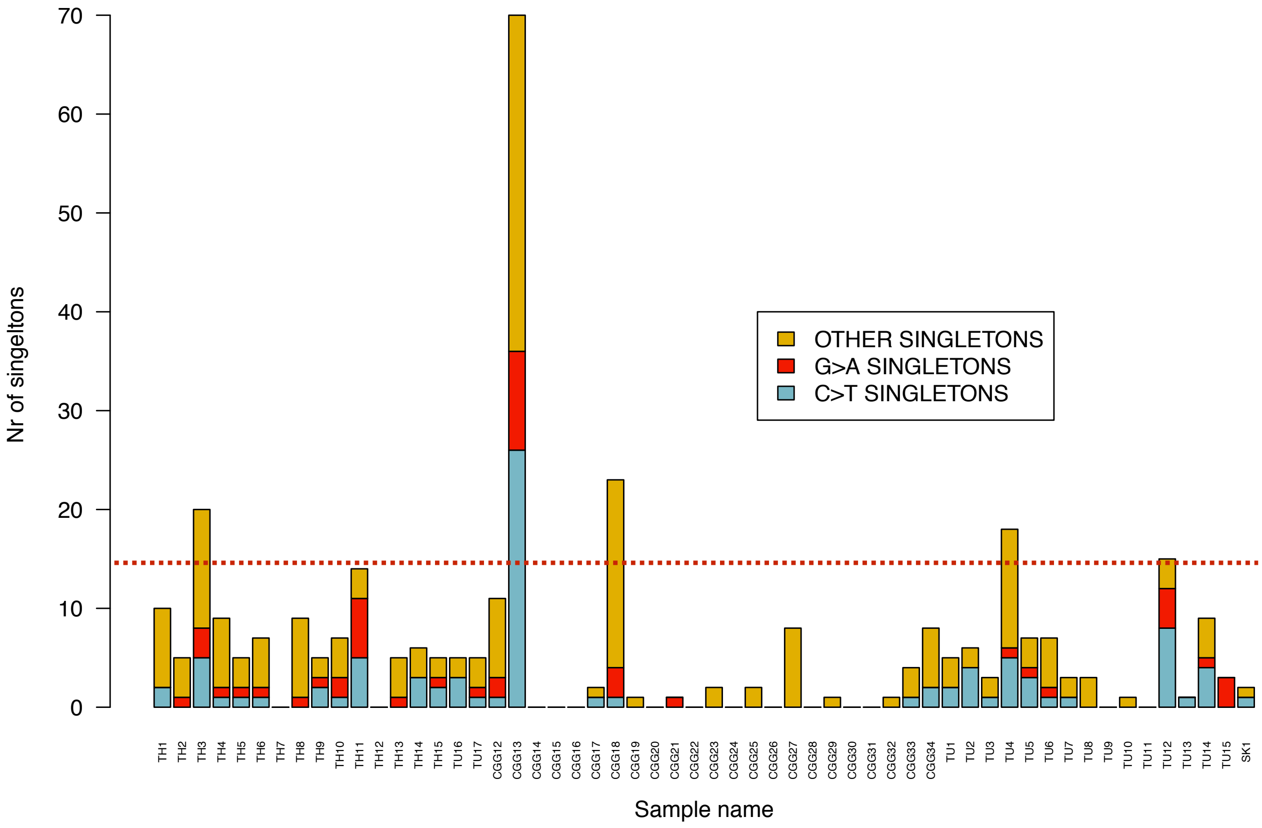

Figure S4: Number of singletons found in each of the sequences. Red represents G to A substitutions, blue represents C to T substitutions and yellow represents transversion substitutions. All ancient sequences that contained more than 0.1% (15 bases) of transition substitutions (C to T or A to G changes) were excluded from all analyses, as an excess of these substitutions indicate large amounts of DNA damage in the form of deamination. The red line represents the cut-off point (15 bases).

MOLECULAR ECOLOGY

Figure S5

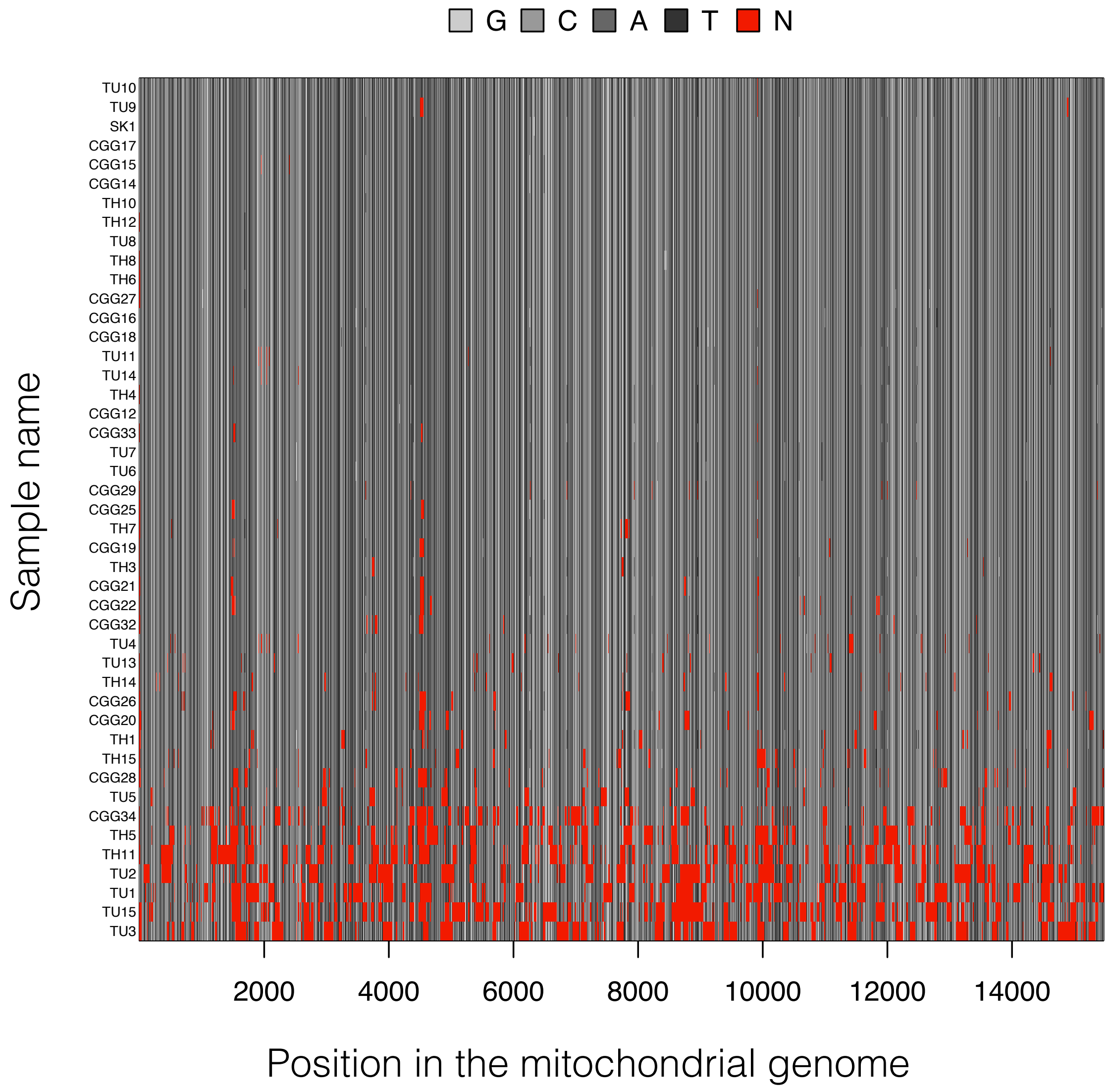

Figure S5: Positions where data is missing (red) for each sample (excluding the control region).

MOLECULAR ECOLOGY

Figure S6

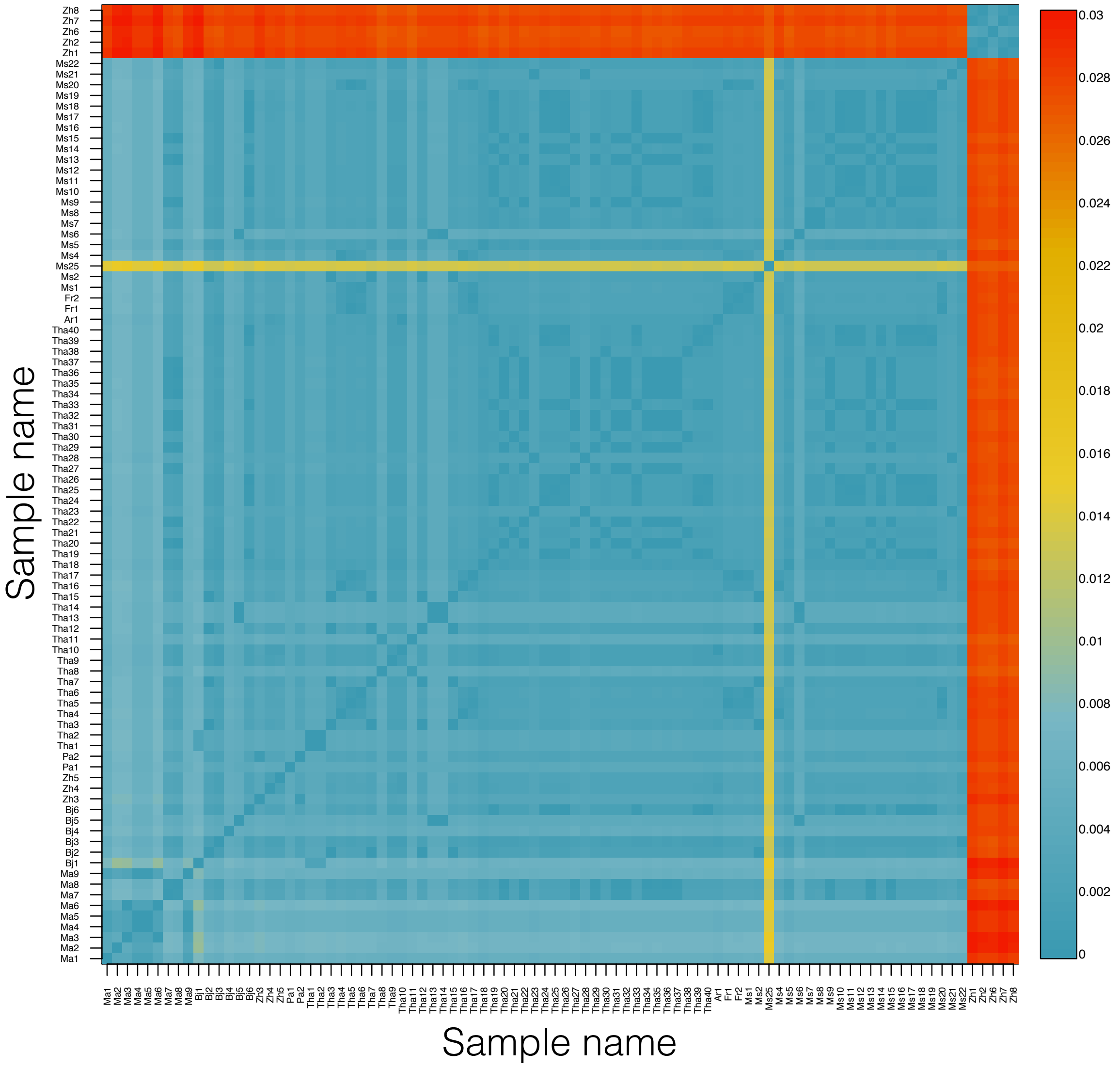

Figure S6: Pairwise genetic similarity (π), measured as proportion of sites that differ between pairs of samples. Values are colour coded, see colour bar on the right, with blue colours corresponding to small π and red colours corresponding to large π.

MOLECULAR ECOLOGY

Figure S7

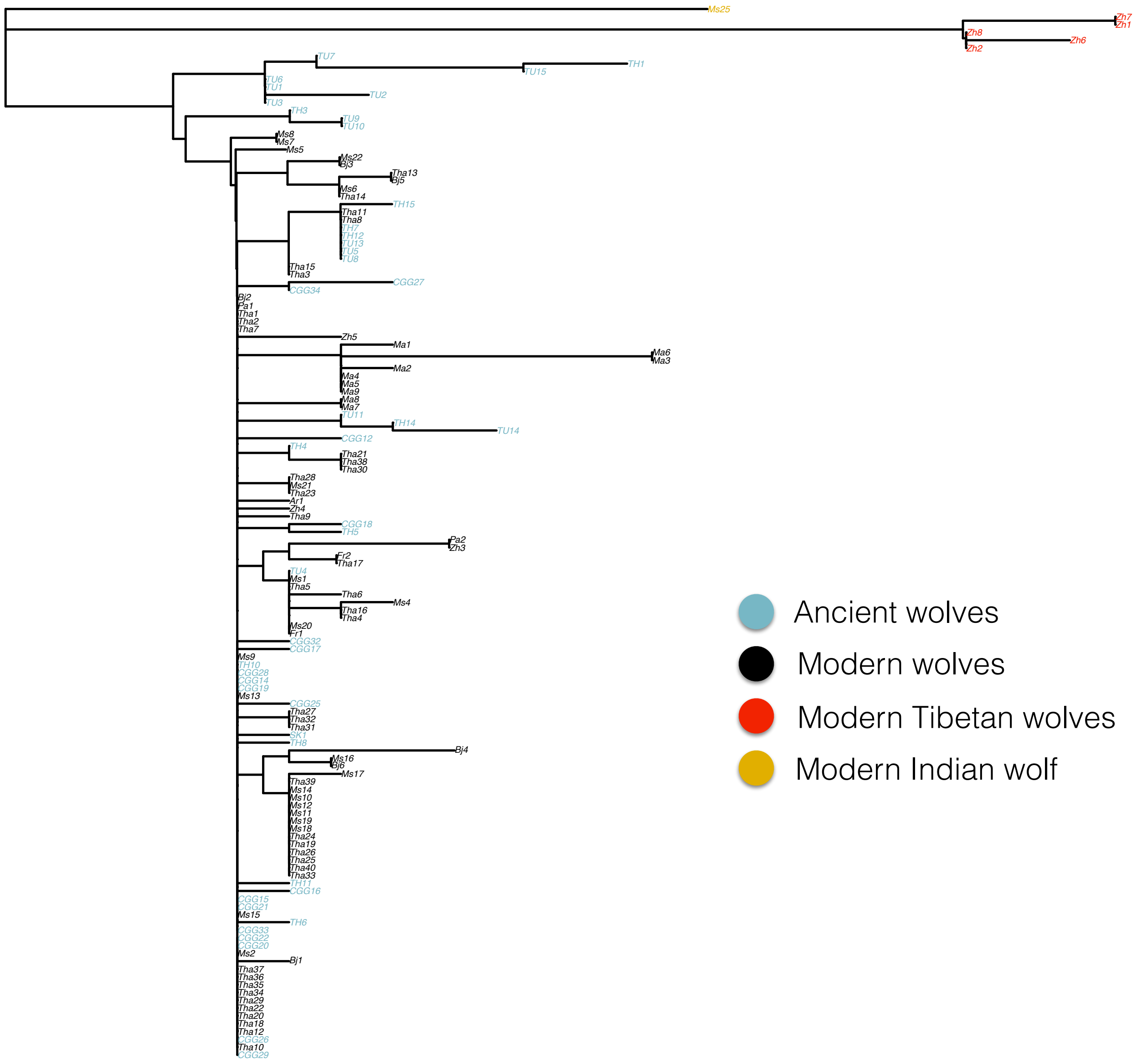

Figure S7: Neighbor-joining tree of all samples based on the pairwise genetic distance (π) matrix, measured as proportion of sites that differ between sequence pairs between samples, calculated using the Ape package in R. Modern northern Eurasian and North American wolves are represented with black labels. Ancient grey (age < 500 years) wolves are represented with blue labels. Modern Tibetan and Indian wolves are represented with red and yellow colours respectively.

MOLECULAR ECOLOGY

Figure S8

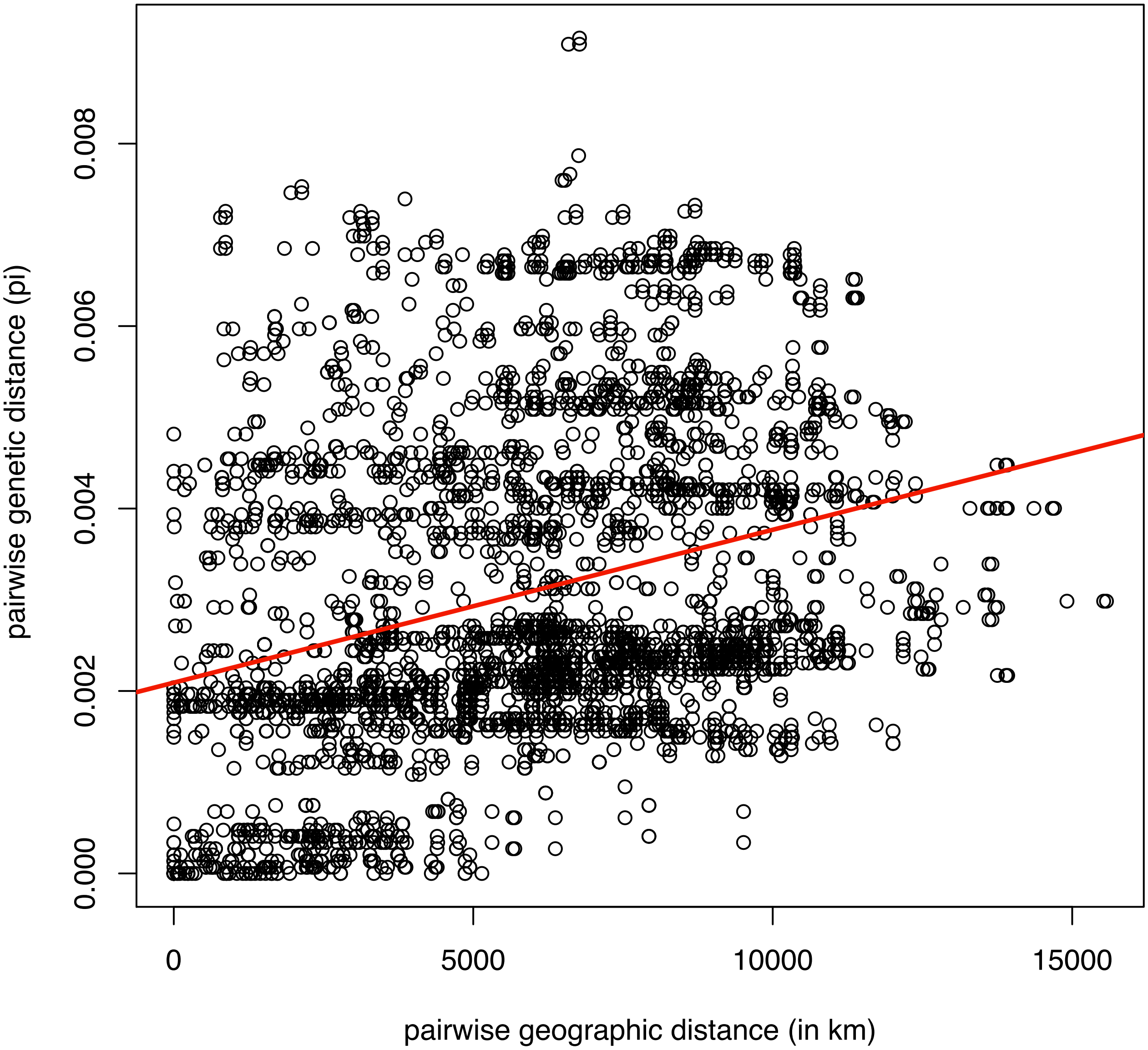

Figure S8: Pairwise geographic distances between all modern North Eurasian and North American wolf samples (N = 84) plotted against the pairwise genetic distances. r= 0.3 (Mantel p < 0.0001).

MOLECULAR ECOLOGY

Figure S9

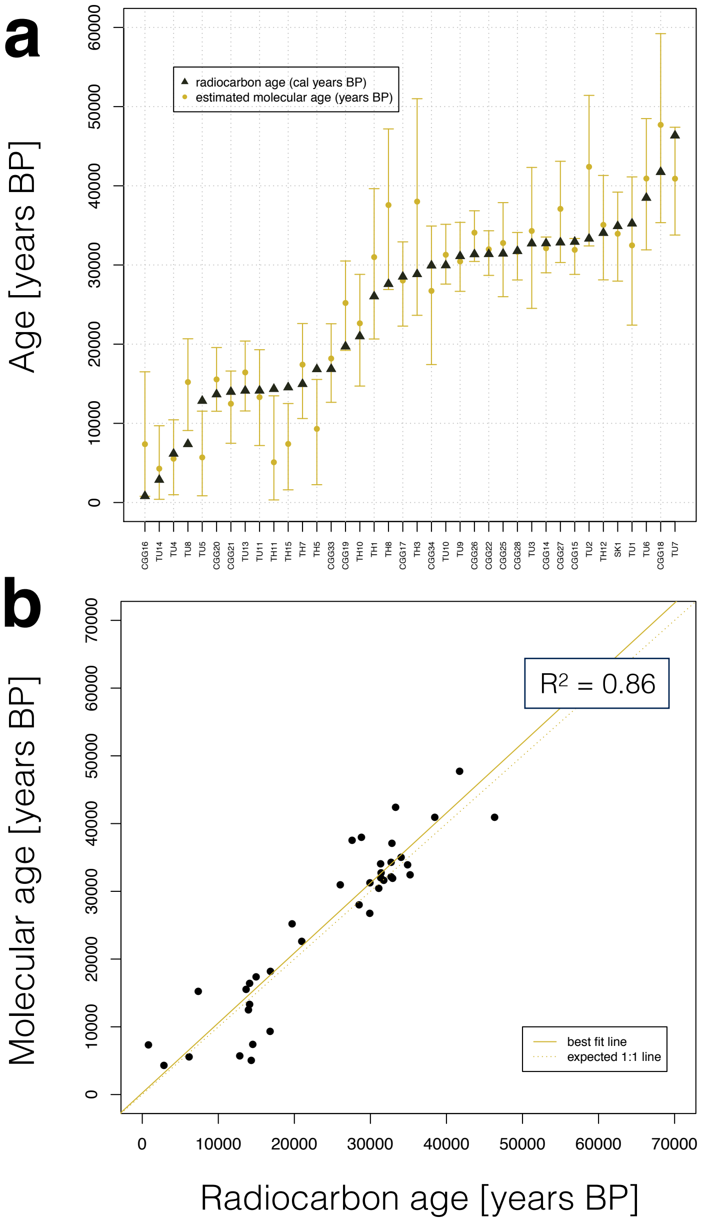

Figure S9: Validation of the molecular dating procedure. (A) Estimated molecular age distributions (yellow lines, yellow dot = median) from the leave-one-out analyses with BEAST and radiocarbon ages (black triangles) for each sample. (B) Linear fit (solid yellow line) between radiocarbon dates and esimated molecular dates from the leave-one-out analyses with BEAST. The dashed yellow line shows the 1:1 correspondence.

MOLECULAR ECOLOGY

Figure S10

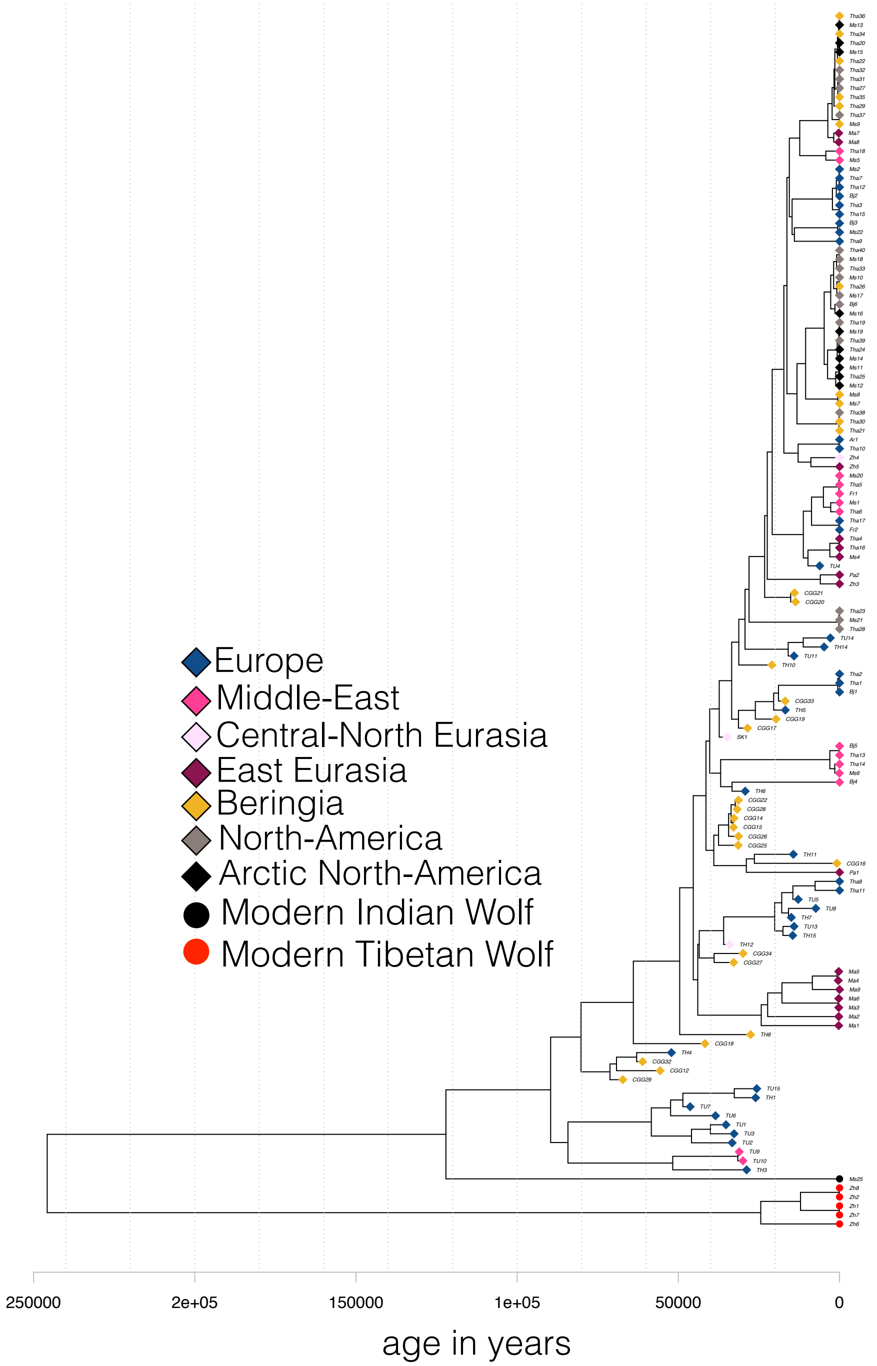

Figure S10: Tip calibrated BEAST tree of all 135 ancient and modern wolf sequences. Circles represent samples excluded from the demographic analyses (wolves from the Himalayas and the Indian subcontinent) and diamonds represent samples included in the demographic, colour coded by geographic regions (demes) used in the analyses.

MOLECULAR ECOLOGY

Figure S11

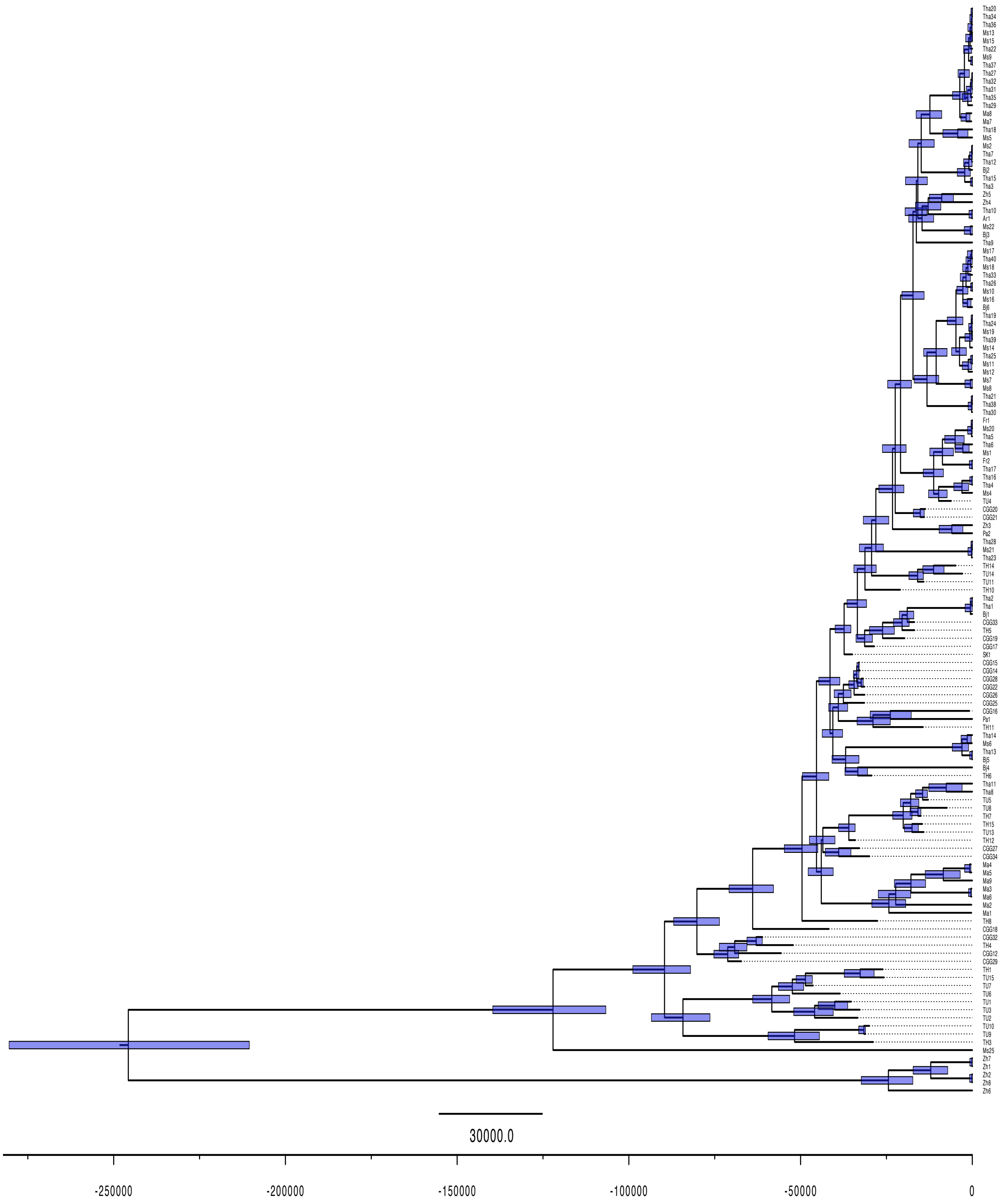

Figure S11: Tip calibrated BEAST tree of all 135 ancient and modern wolf sequences with 95% credibility intervals for all dated nodes indicated by the blue bars.

Figure S12

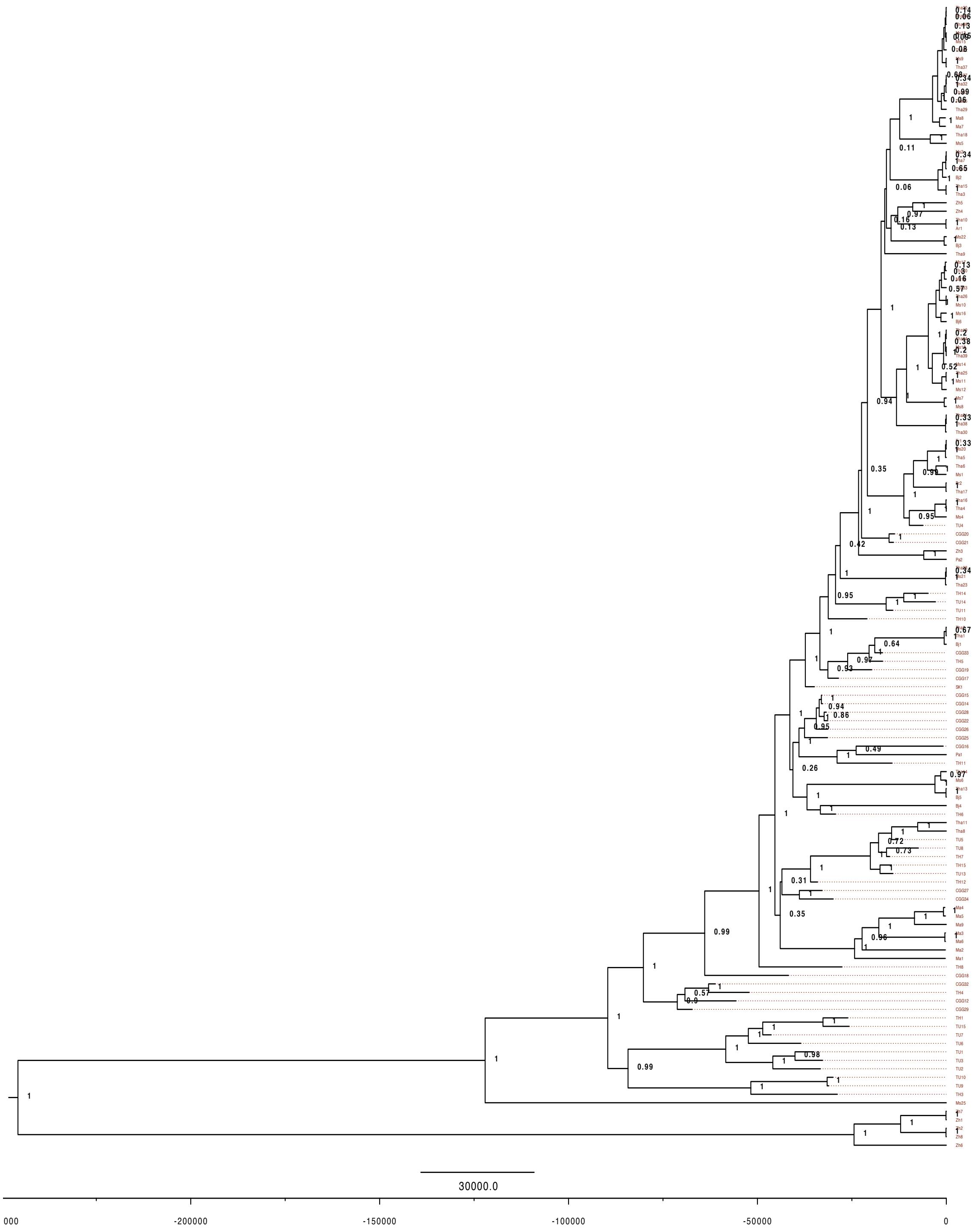

Figure S12: Tip calibrated BEAST tree of all 135 ancient and modern wolf sequences with support values depicted by each node of the tree.

MOLECULAR ECOLOGY

Figure S13

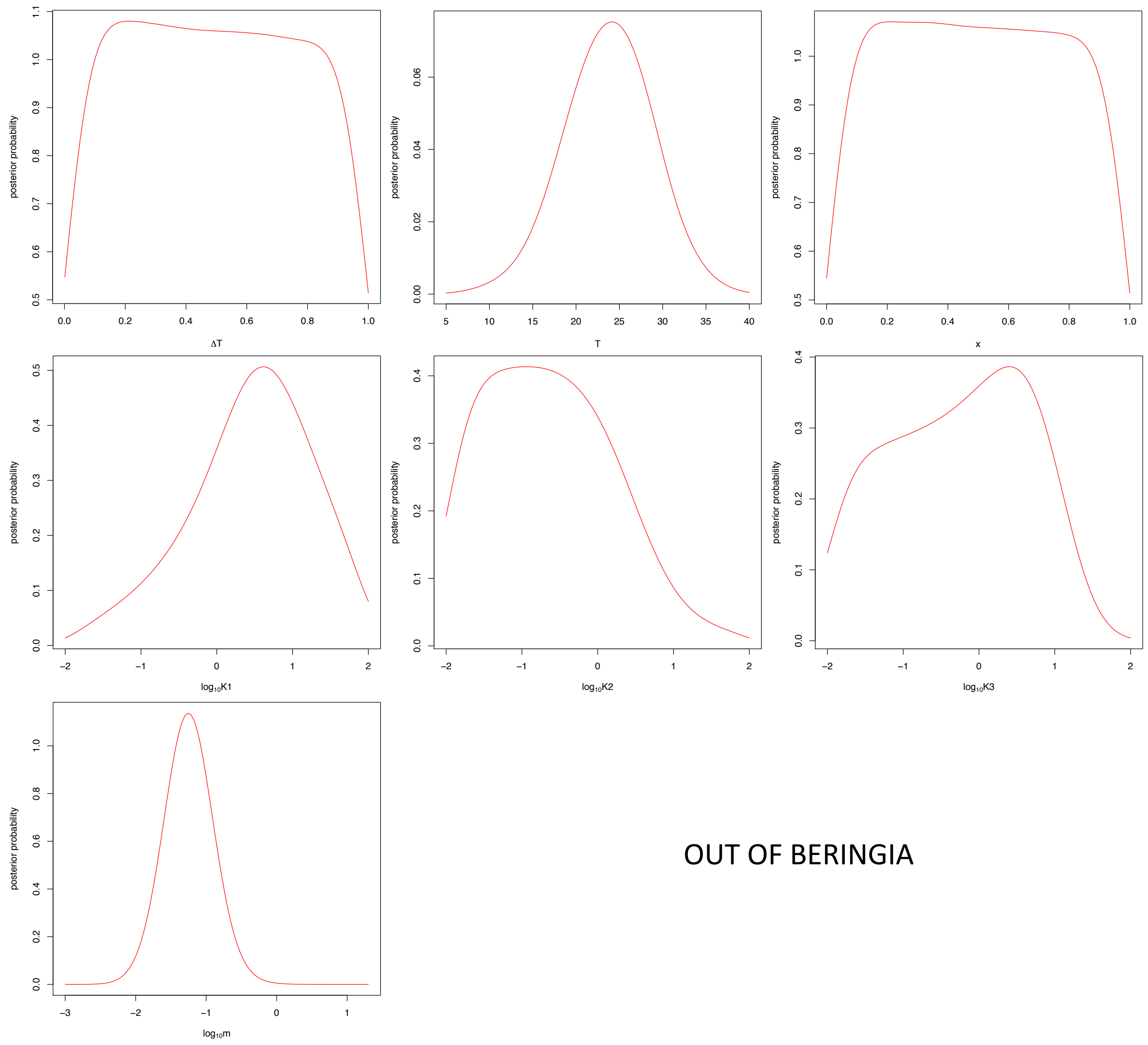

Figure S15: Posterior probability density distribution (on y-axis) for seven estimated parameters (ΔT, T, x, $\log_{10} K_1$, $\log_{10} K_2$, $\log_{10} K_3$, $\log_{10} m$) in the most likely model (Expansion out of Beringia with population size change).

MOLECULAR ECOLOGY

Figure S14

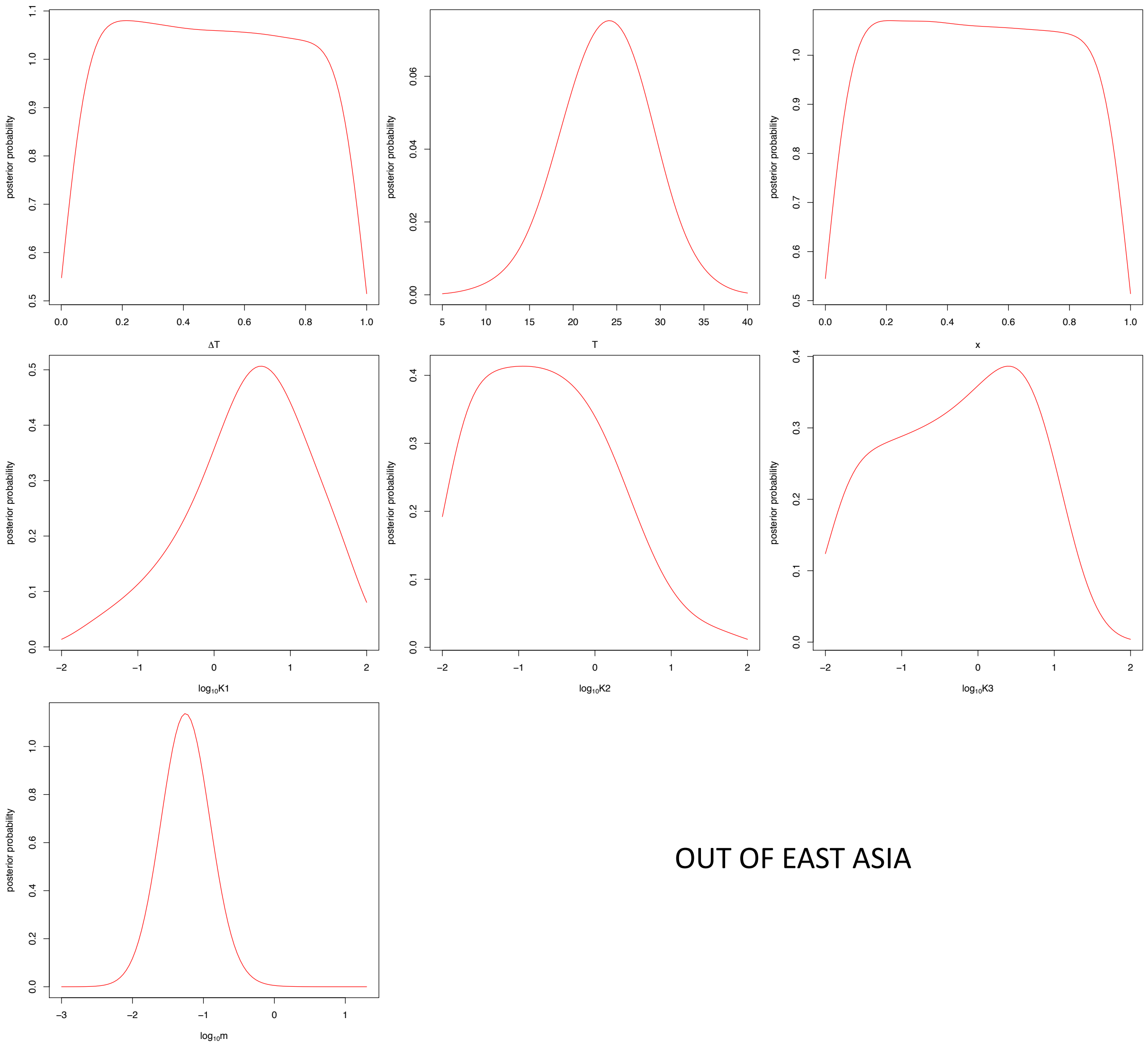

Figure S16: Posterior probability density distribution (on y-axis) for seven estimated parameters (ΔT, T, x, $\log_{10} K_1$, $\log_{10} K_2$, $\log_{10} K_3$, $\log_{10} m$) in the most likely model (Expansion out of East Eurasia with population size change) .
